# An inelastic quadrupedal model discovers four-beat walking, two-beat running, and pseudo-elastic actuation as energetically optimal

**DOI:** 10.1371/journal.pcbi.1007444

**Published:** 2019-11-21

**Authors:** Delyle T. Polet, John E. A. Bertram

**Affiliations:** 1 Department of Biological Sciences, University of Calgary, Calgary, Alberta, Canada; 2 Department of Cell Biology and Anatomy, Cumming School of Medicine, University of Calgary, Calgary, Alberta, Canada; Simon Fraser University, CANADA

## Abstract

It is widely held that quadrupeds choose steady gaits that minimize their energetic cost of transport, but it is difficult to explore the entire range of possible footfall sequences empirically. We present a simple model of a quadruped that can spontaneously produce any of the thousands of planar footfall sequences available to quadrupeds. The inelastic, planar model consists of two point masses connected with a rigid trunk on massless legs. It requires only center of mass position, hind and forelimb proportions and a stride-length to speed relationship as input. Through trajectory optimization of a work and force-rate cost, and a large sample of random initial guesses, we provide evidence for the global optimality of symmetrical four-beat walking at low speeds and two beat running (trotting) at intermediate speeds. Using input parameters based on measurements in dogs (*Canis lupus familiaris*), the model predicts the correct phase offset in walking and a realistic walk-trot transition speed. It also spontaneously reproduces the double-hump ground reaction force profile observed in walking, and the smooth single-hump profile observed in trotting. Actuation appears elastic, despite the model’s lack of springs, suggesting that spring-like locomotory behaviour emerges as an optimal tradeoff between work minimization and force-rate penalties.

## Author summary

Why do quadrupedal mammals move in such consistent ways, when so many options are available? We tackled this problem by determining energetically-optimal gaits using a simple computational model of a four-legged animal. The model can use virtually any pattern of movement (physics-permitting!) but selects movement strategies observed in nature as energetically optimal. The similarities between the computer-based predictions and natural animal movement are striking, and suggest mammals utilize movement strategies that optimize energy use when they move.

## Introduction

When unconstrained at a given speed, members of a quadrupedal species will generally select a common gait, which is seldom unique to that species alone [1–4]. The consistency of gait choice is remarkable, given how many alternatives exist. McGhee and Jain [5] noted that while 5040 unique footfall sequences are available to quadrupeds, only 21 varieties had been reported by the mid-1970s (when the authors assayed the literature). Within these varieties, some very consistent patterns emerge. With few exceptions, mammals choose a lateral or diagonal sequence (four-beat) walk at slow speeds, a running trot (two-beat) at intermediate speeds, and a gallop (asymmetric) at high speeds [6, 7]. Before the trot-gallop transition, healthy mammals use highly symmetrical gaits, with foot touchdown and liftoff on the left side of the body being repeated exactly half a stride later on the right side [1, 8, 9]. Why should the same patterns repeatedly emerge among quadrupedal mammals, despite the incredible morphological diversity and near limitless gait possibilities?

One view is that spring-like leg actuation dominates natural locomotion, with ground contact forces being proportional to the length change of the leg [10, 11], and that this pattern of actuation leads to certain gaits being used. In passive spring-based models, spring-like elastic actuation can generate many gaits, depending on which resonances or “oscillation modes” are exploited. For example, bipedal running appears to use one compression and extension cycle, generating a “single-hump” ground reaction force (GRF) profile, while bipedal walking involves two cycles with double-hump GRFs [12]. The same principles can generate gaits observed in quadrupeds [13], including the four-beat walk and two-beat trot.

Another view is that the gaits utilized by quadrupeds at a particular speed are those that minimize metabolic energy expenditure [1–4, 14]. Since locomotion is an energy-intensive activity, it is reasonable to suppose that animals have evolved some means to lower the energetic cost of locomotion. Indeed, humans seem remarkably sensitive to cost of transport, or energetic expenditure per unit weight per unit distance, even shifting their gait in response to unusual stimuli in accordance with energetic cost mitigation [15–17]. It is more difficult to train animals to perform unusual gait tasks, but Hoyt and Taylor [14] showed that horses will exhibit increased metabolic cost of transport if they perform walking, trotting or galloping outside their natural speed ranges, and tend to choose speeds that minimize cost of transport within each gait.

These two views, while not necessarily contradictory, are surprisingly difficult to reconcile. One approach is to suppose that spring-like actuation takes advantage of elastic storage and return [18]. However, estimates of the contribution of elasticity to gait economy vary widely, from 3 to 64% of the net metabolic cost in quadrupedal running, depending on gait, organism and size [19]. If spring-like gaits can have small elastic savings, why should the elastic components determine the control strategy?

A possibility for reconciling these two views is the concept of pseudo-elastic collisions [20, 21]. That is, by minimizing work, a spring-like behaviour can emerge from an inelastic system. Ruina *et al*. [20] examined a wide variety of cases, modelling stance as an active collision through the leg with the ground, and showed that muscular work is minimized when it is performed only along the leg axis. A consequence is that the initial and final contact angle are opposite– as one would expect if contact behaved like an elastic collision.

Through trajectory optimization of a simple inelastic biped model, Srinivasan and Ruina [22] showed that work-minimizing walking and running gaits exhibit pseudo-elastic double-hump and single-hump ground reaction forces respectively. However, these profiles exhibited impulsive peak forces, as is typical for work-minimizing inelastic locomotion [23], unlike the smooth ground-reaction forces observed in natural gait.

There is increasing evidence from the human physiology literature that muscle activation costs, in addition to muscular work, are an important consideration in gait strategy. That is, metabolic costs increase when muscular forces change quickly, even if work is held constant [24–26]. While the physiological mechanism is currently unknown, rate penalties explain much about gait selection, particularly the stride length and duty factor *vs*. speed relationship [27–29]. In particular, smooth ground reaction forces can emerge from an inelastic bipedal model when a force-rate penalty is optimized in addition to work [30]

Work-minimizing quadrupedal gaits have not been examined in as much detail as the bipedal case. Xi *et al*. [31] approached the issue by including mechanisms of energetic loss in their planar quadrupedal model (collisions and damping), as well as series-elastic actuators (SEAs). They found that a four-beat walk is work minimizing at slow speeds and a two-beat run (trotting or pacing) minimizes work at high speeds. They pointed to the exploitation of oscillatory modes to explain why their model exhibited smooth double-hump ground reaction force (GRF) profiles in walking and single hump GRFs in running. However, without removing the elastic elements, it is not straightforward to determine whether *pseudo-elastic actuation alone* might be energetically optimal.

The relative complexity of their model (eight SEAs, nine rigid elements, and damping and collision dynamics) makes it difficult to determine which aspects of the model are responsible for the emergent behaviour and to explore a wide sample of footfall patterns. It is possible that other unexplored footfall sequences might be energetically optimal for quadrupeds.

To what extent can a simple, minimally constrained energy-optimization model without springs predict gait choice in quadrupeds? Do natural gaits emerge from such a simplified model and are they energy minimizing? Does a force-rate cost substantially enhance empirical agreement? We explore these questions with trajectory optimization on a planar, two-point-mass model that can perform any of the footfall sequences available to quadrupeds. We compare the simulation results to empirical data on dogs only (due to the wealth of data available), but we anticipate the results are more broadly applicable across quadrupedal mammals.

## Methods

### Generalized model of a quadruped

We use a simplified model of a quadruped based on the bipedal model in [22], but extended to four legs. A rigid trunk connects two point masses. Each point mass connects to two massless limbs that can extend or contract prismatically. The quadruped lives in the sagittal plane and experiences downward gravitational acceleration *g* while translating at an average horizontal speed *U*.

Five parameters are derived from empirical measurements, and provided to the model. These are the stride length as a function of speed *D*(*U*) (giving the stride period *T*), forequarter relative mass $m_{F}^{\prime }=m_{F}/m$ (where *m* is body mass), shoulder-to-hip length *l*_*B*_, and hind and forelimb lengths relative to body length ($l_{H\text{max}}^{\prime }$ and $l_{F\text{max}}^{\prime }$ respectively). We normalize lengths by *l*_*B*_, time by *T*, masses by *m* and forces by *mg*. These result in a dimensionless time constant

$$\hat{T}\equiv T\sqrt{g/l_{B}}$$ (1)

which appears in a number of equations. Normalized variables are given the prime superscript (⋅)′, and normalized time goes from *t*′ = 0 to 1. Other conventions (and all symbols with their meanings) are shown in Table 1.

**Table 1:** A list of symbols used in this paper and their meanings.

Symbol	Meaning
*θ*	Trunk pitch angle (rad)
$c_{1}^{\prime }$	Normalized force-rate penalty coefficient (*l*_*B*_ *T* (*mg*))*
$c_{1D}^{\prime }$	Normalized force-rate penalty coefficient for the Dalmation case (*l*_*B*_ *T* (*mg*))*
*c*_2_	Penalty coefficient for *p*_*ijk*_ *q*_*ijk*_ complementarity violation*
*c*_3*a*_, *c*_3*b*_, *c*_3*c*_	Penalty coefficients for *s*_*aijk*_, *s*_*bij*_ and *s*_*cijk*_ minimization*
COM	Centre of mass
CoT	Cost of Transport
DF	Duty factor
*D*	Stride length (m)* ^†^
$f_{ijk}^{\prime }$	Footfall postions (*l*_*B*_)^‡^
$F_{ijk}^{\prime }\left(t^{\prime }\right)$	Limb force (*mg*)^‡^
FL	Fore Lag
**F**_Fm_, **F**_Hm_	Mean fore or hindlimb force vectors (N)^†^
GRF	Ground reaction force
HL	Hind Lag
*I*	Trunk pitch moment of inertia (kg m^2^)
$I^{\prime }=m_{F}^{\prime }m_{H}^{\prime }$	Two-point-mass moment of inertia ($ml_{B}^{2}$)*
$\hat{I}\equiv 4I/\left(ml_{B}^{2}\right)$	Dimensionless pitch moment of inertia
*k*′	Apparent stiffness $\left(\Delta F_{ijk}^{\prime }/\left(\Delta l_{ijk}^{\prime }/l_{i\text{max}}^{\prime }\right)\right)$ ^‡^
*l*_*B*_	Shoulder to hip length (m)* ^†^
$l_{ijk}^{\prime }\left(t^{\prime }\right)$	Limb length (*l*_*B*_)^‡^
$l_{F\text{max}}^{\prime }$	Shoulder to manus length in standing (*l*_*B*_)* ^†^
$l_{H\text{max}}^{\prime }$	Hip to pes length in standing (*l*_*B*_)* ^†^
LHTD	Touchdown of the left-hind limb
*m*	Total body mass (kg)* ^†^
$m_{F}^{\prime }$	Forequarter mass (*m*)* ^†^
$m_{H}^{\prime }$	Hindquarter mass (*m*)* ^†^
$p_{ijk}^{\prime }\left(t^{\prime }\right)$	Positive limb power (*mgl*_*B*_/*T*)^‡^
PL	Pair Lag
$q_{ijk}^{\prime }\left(t^{\prime }\right)$	Negative limb power (*mgl*_*B*_/*T*)^‡^
$r_{F}^{\prime }\left(t^{\prime }\right)$	Displacement vector from COM to shoulders (*l*_*B*_)^‡^
$r_{H}^{\prime }\left(t^{\prime }\right)$	Displacement vector from COM to hips (*l*_*B*_)^‡^
$s_{aijk}\left(t^{\prime }\right),\;s_{bij}\left(t^{\prime }\right),\;s_{cijk}\left(t^{\prime }\right)$	Complementarity relaxation parameters^‡^
SEA	Series elastic actuator
*t*′	Time during stride (*T*)^‡^
*T*	Stride period (s)* ^†^
$\hat{T}$	Dimensionless time constant ($T\sqrt{l_{B}/g}$)* ^†^
${\hat{T}}_{D}$	Dimensionless time constant for the Dalmation test case ($T\sqrt{l_{B}/g}$)* ^†^
*T*_*n*_	Leg natural pendulum period (s)^†^
*U*	Mean fore-aft speed (m/s)* ^†^
**x**′(*t*′) = (*x*′, *y*′,0)^⊤^	CoM displacement (*l*_*B*_)^‡^
${\dot{x}}^{\prime }\left(t^{\prime }\right)={\left({\dot{x}}^{\prime },{\dot{y}}^{\prime },0\right)}^{⊤}$	CoM velocity (*l*_*B*_/*T*)^‡^
	**Special operators and subscripts**
(⋅)′	Normalized variable
(⋅)^⊤^	Matrix transpose
*i* ∈ {*F*, *H*}	Subscript: Fore, Hind
*j* ∈ {*R*, *L*}	Subscript: Right, Left
*k* ∈ {*T*, *L*}	Subscript: Trailing contact, Leading contact
$\hat{x},\;\;\hat{y},\;\;\hat{z}$	Fore-aft, vertical and medio-lateral unit vectors

Symbols are arranged alphabetically. Variables that are specifically model inputs or outputs are noted, as are those which are only derived empirically.

*Inputs to Optimization

^†^Empirically Derived Only

^‡^Outputs from optimization

The vectors from the centre of mass (COM) to the fore and hindquarters are given, respectively, as

$$r_{F}^{\prime }=m_{H}^{\prime }{\left(\begin{array}{ccc} \cos\theta , & \sin\theta , & 0 \end{array}\right)}^{⊤},$$ (2)

$$r_{H}^{\prime }=-m_{F}^{\prime }{\left(\begin{array}{ccc} \cos\theta , & \sin\theta , & 0 \end{array}\right)}^{⊤}.$$ (3)

Note that we denote cartesian vectors with a bold font. Since the model is sagittal, the *z* (out of plane) coordinate is constrained to be zero. The trunk is a rigid link with two point masses at either end, giving a normalized moment of inertia of $I^{\prime }=m_{F}^{\prime }\cdot m_{H}^{\prime }$ (where $m_{H}^{\prime }$ is the relative mass supported by the hindlimbs). The trunk’s COM position is **x** = (*x*, *y*,0)^⊤^, and its orientation relative to the horizontal is *θ*.

**Fig 1:**
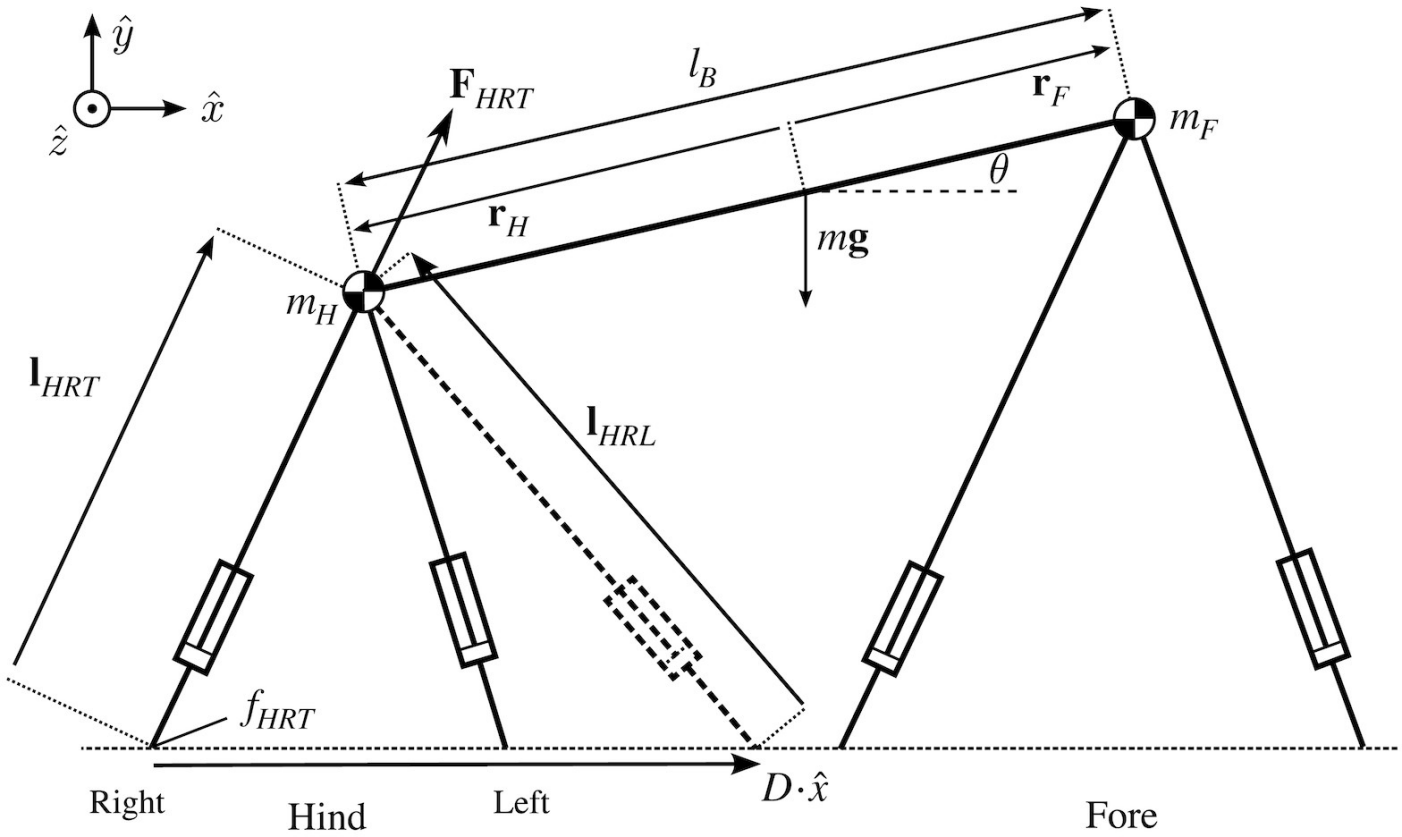
The simple quadrupedal model used in this paper. Two point masses sit on massless legs that can extend and contract. The point masses are connected with a rigid trunk. Five morphological parameters are inputs: the mass of the fore and hindquarters (*m*_*F*_ and *m*_*H*_, respectively), the trunk length (*l*_*B*_), and the maximum length of the fore and hindlimbs (*l*_*Fmax*_ and *l*_*Hmax*_, respectively). Two kinematic variables are also used as inputs: the stride length (*D*) and the average horizontal speed (*U*). From these seven constant inputs, trajectory optimization determines footfall positions (*f*_*ijk*_, where *i*, *j* and *k* refer to Fore-Hind, Right-Left and {Trailing contact}-{Leading contact}, respectively) and over twenty temporal variables, including ground reaction forces and their rate of change (*F*_*ijk*_ and ${\dot{F}}_{ijk}$), center of mass position and velocity (**x** and $\dot{x}$) and body pitch angle and angular velocity (*θ* and $\dot{\theta }$). **r**_*F*_ and **r**_*H*_ are the displacement vectors from the COM to forequarters or hindquarters, respectively. Other notation conventions are described in Table 1.

**Fig 2:**
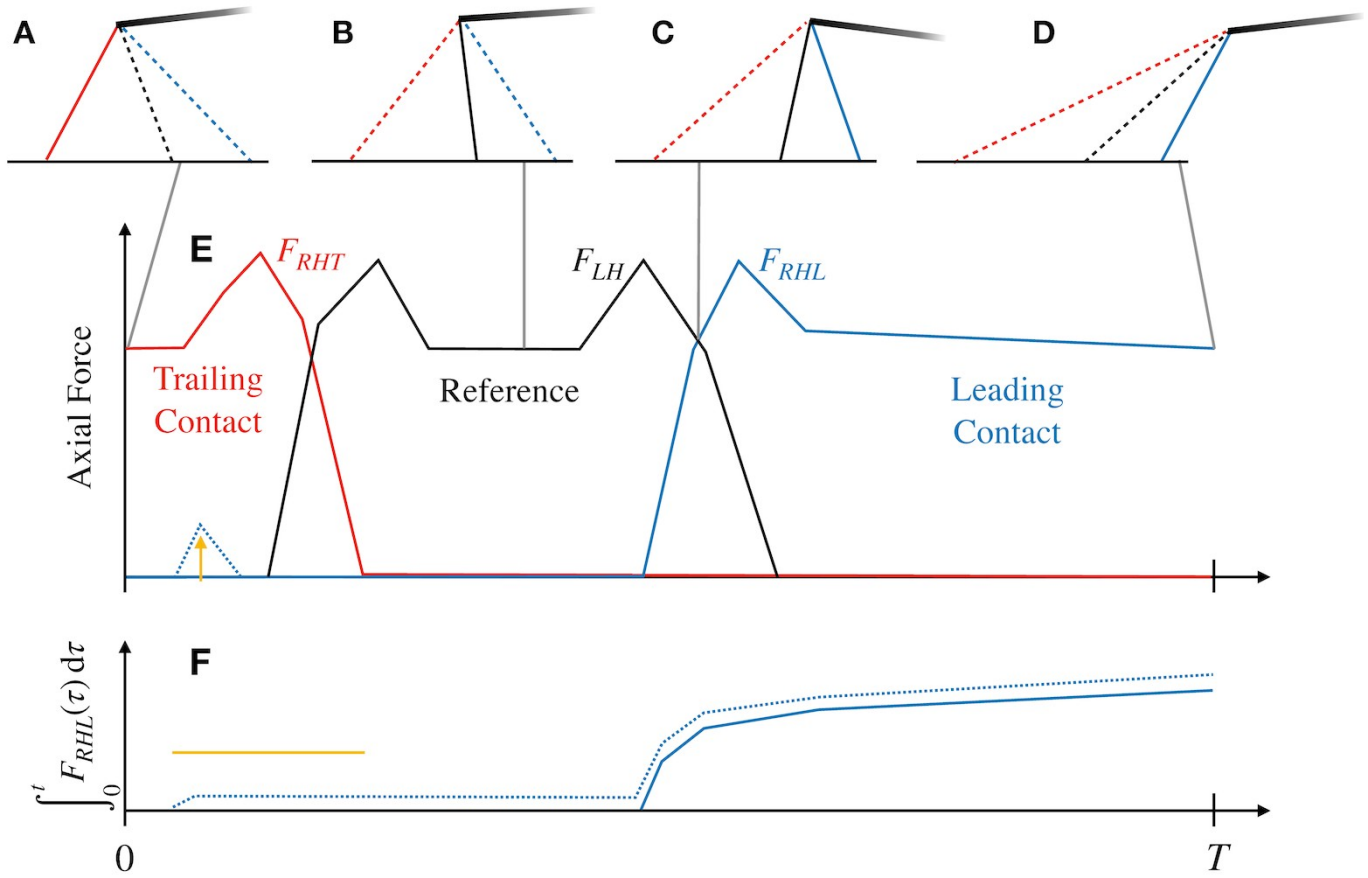
The strategy for determining stepping sequence. (A-D) The configuration of the hindlimbs are shown at four points in the stride cycle, with right limbs in colour and the left (reference) in black. In this modelling approach, limbs are always “connected” to the body, but are either producing force (solid lines) or not (dashed lines) (A) At *t* = 0, the right limb acting through trailing contact (red) can produce an axial force *F*_*RHT*_(0) ≥ 0, but the reference (black) and limb acting through leading contact (blue) cannot produce force (*F*_*LH*_ = *F*_*RHL*_ = 0). (B) At some point in the cycle, the right limb is in swing, with *F*_*RHT*_ = *F*_*RHL*_ = 0. (C) The force through leading contact exceeds zero and causes *F*_*RHT*_ = 0 for the remainder of stride. (D) The right hind limb starts and ends in the same configuration, but one stride length forward, acting through leading contact. (E) Forces shown during the stride. The right limb may start and end the stride in stance, so is modelled with “trailing contact” and “leading contact” forces. The reference limb starts and ends the stride in swing. (F) As soon as *F*_*RHL*_ becomes positive, its time integral from *t* = 0 becomes positive for the rest of the stride (since *F*_*ijk*_(*t*) ≥ 0∀*t*). The constraint Eq 8 then ensures that *F*_*RHT*_ cannot be nonzero again. The dotted line in E shows a violation of Eq 8 for some portion of stance (yellow line).

We describe axial limb forces through the states *F*_*ijk*_(*t*) ≥ 0, where the subscripts *i*, *j*, and *k* denote {Fore, Hind}, {Right, Left}, and {Trailing contact, Leading contact} respectively (*e.g*. *F*_*HRL*_ is the force of the Hind-Right limb through Leading contact). Trailing contact positions are given as $f_{ijT}^{\prime }$, while leading contact positions are $f_{ijL}^{\prime }\equiv f_{ijT}^{\prime }+D^{\prime }$ (Fig 1). The four footfall positions $f_{ijT}^{\prime }$ are decision variables for the problem. Since three limbs act through two contact positions, while one is our “reference” and acts through a single contact position (Fig 2E), we require seven states to describe axial limb forces.

The limb length vectors are

$$\begin{array}{c} l_{ijk}^{\prime }=x^{\prime }+r_{i}^{\prime }-f_{ijk}^{\prime }\hat{x} \end{array}$$ (4)

and the limb forces are

$$\begin{array}{c} F_{ijk}^{\prime }\left(t\right)=F_{ijk}^{\prime }\frac{l_{ijk}^{\prime }}{l_{ijk}^{\prime }}. \end{array}$$ (5)

Here $l_{ijk}^{\prime }=\left|\right|l_{\text{ijk}}^{\prime }\left|\right|$. As the body consists of only one rigid link, the dynamics are simply

$$\begin{array}{c} \begin{array}{cc} {\ddot{x}}^{\prime } & =\left(-\hat{y}+\sum_{ijk}F_{ijk}^{\prime }\right){\hat{T}}^{2}\\ \ddot{\theta } & =\frac{\sum_{ijk}r_{i}^{\prime }\times F_{ijk}^{\prime }}{I^{\prime }}{\hat{T}}^{2} \end{array} \end{array}$$ (6)

and are the first set of path constraints for the model.

We do not wish to impose the stepping sequence, instead allowing it to be part of the optimization problem. In effect, all limbs are connected to the ground at all times (Fig 2A–2D); however, leg forces may only be generated when limbs are sufficiently short (*i.e*. less than measured limb length in standing):

$$\begin{array}{c} \left(l_{i\text{max}}^{\prime }-l_{ijk}^{\prime }\right)F_{ijk}^{\prime }\geq 0 \end{array}$$ (7)

We must also ensure that limbs act through trailing contact *before* leading contact (Fig 2E and 2F), and so impose the following path constraint:

$$\begin{array}{c} F_{ijT}^{\prime }\;\int_{0}^{t^{\prime }}\;\;\;F_{ijL}^{\prime }\left(\tau \right)d\tau =0. \end{array}$$ (8)

Once a solution is found, we can examine the ground reaction forces to assess contact. A limb is considered to be in contact with the ground if and only if its ground reaction force is nonzero. With this method, we do not need to specify contact sequence *a priori*– an advantage over the multiple shooting method used by [31]– but with the disadvantage that solutions exhibit disjunctive behaviour at ground contact. This requires special treatment and makes the problem more difficult to solve. The relative simplicity of our model makes our implementation tractable, despite the disadvantage.

We add two more sets of path constraints. One ensures the torso is above the ground:

$$\begin{array}{c} \left(x^{\prime }+r_{i}^{\prime }\right)\cdot \hat{y}>0,\;\;i=\left\{F,H\right\}, \end{array}$$ (9)

Another set of constraints keeps limbs oriented ventrally by ensuring that torques about the COM generated by forelimb forces are positive, while those generated by hindlimb forces are negative:

$$\begin{array}{c} \begin{array}{cc} r_{F}^{\prime }\times F_{Fjk}^{\prime } & \geq 0,\\ -r_{H}^{\prime }\times F_{Hjk}^{\prime } & \geq 0. \end{array} \end{array}$$ (10)

The following endpoint constraints are prescribed to ensure a steady gait,

$$\begin{array}{ccc} {\left(y^{\prime },\theta ,{\dot{x}}^{\prime },{\dot{y}}^{\prime },\dot{\theta }\right)}^{⊤}\;{|}^{t^{\prime }=0}={\left(y^{\prime },\theta ,{\dot{x}}^{\prime },{\dot{y}}^{\prime },\dot{\theta }\right)}^{⊤}\;{|}^{t^{\prime }=1} & & \text{(Kinematics}\;\text{are}\;\text{periodic),} \end{array}$$ (11)

$$\begin{array}{ccc} F_{ijL}^{\prime }\left(1\right)-F_{ijT}^{\prime }\left(0\right)=0 & & \text{(Forces}\;\text{are}\;\text{continuous),} \end{array}$$ (12)

along with the following endpoint bounds:

$$\begin{array}{cc} x^{\prime }\left(0\right) & =0, \end{array}$$ (13)

$$\begin{array}{cc} x^{\prime }\left(1\right) & =D^{\prime }, \end{array}$$ (14)

$$\begin{array}{cc} F_{ijL}^{\prime }\left(0\right)=F_{ijT}^{\prime }\left(1\right) & =0. \end{array}$$ (15)

Two integral constraints are also prescribed, ensuring that the vertical and fore-aft impulse are equal on the left and right side:

$$\begin{array}{c} \int_{0}^{⊤}\;\sum_{i,k}\;F_{iLk}^{\prime }-\sum_{i,k}F_{iRk}^{\prime }dt=0. \end{array}$$ (16)

While the model is 2D, these constraints have a basis in three dimensions. If we assume constant moment arms on the left and right side, then violating Eq 16 would cause the animal to roll or yaw. Including the integral constraints lowered solve time, while removing these constraints did not seem to change the optimal solution.

### Cost function

Our hypothesis is that quadrupedal mammals choose whichever steady gait minimizes metabolic cost per unit distance. The primary contributor to energetic cost is limb work, the combined positive and negative work performed by each limb:

$$\begin{array}{c} J_{w}=\int_{0}^{⊤}\;\sum_{i,j,k}\left|F_{ijk}\left(t\right){\dot{l}}_{ijk}\left(t\right)\right|dt. \end{array}$$ (17)

Note that we make no distinction between positive and negative muscle work efficiencies. While these efficiencies can alter optimal solutions in unsteady gaits [32], in steady gaits on level ground an equal amount of positive and negative work must be performed. Therefore, in the absence of other forms of dissipation (like foot-ground collisions or viscous effects) or asymmetric elastic energy recovery, the relative efficiencies of positive and negative work do not change the optimal solution.

The secondary cost is that of a force-rate penalty. Since the exact choice of rate penalty has little effect on the agreement with empirical data [30] (some fitting is required in any case, since the mechanism is unknown), we used a squared cost in force rate,

$$\begin{array}{c} J_{r}=\int_{0}^{⊤}\;c_{1}{\dot{F}}_{ijk}^{2}dt, \end{array}$$ (18)

where *c*_1_ is a fitting parameter. A side benefit of the force rate cost is that it smooths the otherwise impulsive solutions that result from work-minimization [23, 30, 32].

The resulting objective of the optimization (in the absence of augmentation for complementarity minimization), reflecting the metabolic cost of transport in the absence of a basal metabolic rate, is

$$\begin{array}{c} J_{\text{tot}}=\frac{J_{w}+J_{r}}{mgD}, \end{array}$$ (19)

where *D* is the stride length.

#### Numerically well-conditioned trajectory optimization problem

Three numerical issues arise in the ideal implementation. The first is a non-smooth objective due to the absolute-value function in its integrand (Eq 17). The second is due to disjunctive behaviour arising from complementarity conditions. The final issue is due to poor scaling, arising because several variables have vastly different magnitudes. Each issue can be ameliorated by reformulating the problem.

Non-smooth functions in optimal control problems can be reformulated using slack variables [33]. Two slack variables are required for every instance of the absolute value function in Eq 17; thus 14 new controls are introduced: *p*_*ijk*_(*t*) ≥ 0 and *q*_*ijk*_(*t*) ≥ 0, representing positive and negative limb power respectively.

In addition, new path constraints are required, one set for redefining the operand of the absolute value function,

$$\begin{array}{c} F_{ijk}^{\prime }\cdot {\dot{l}}_{ijk}^{\prime }-p_{ijk}^{\prime }+q_{ijk}^{\prime }=0, \end{array}$$ (20)

and another for enforcing complementarity of the slack variables

$$\begin{array}{c} p_{ijk}^{\prime }\;q_{ijk}^{\prime }=0. \end{array}$$ (21)

*p*_*ijk*_ + *q*_*ijk*_ then replace $|F_{ijk}\left(t\right){\dot{l}}_{ijk}|$ in Eq 17.

Unfortunately, Eq 21 is a complementarity condition, as are several other constraints (Eqs 7, 8 and 10) which require special treatment [33, 34]. One way to manage these conditions is to introduce “relaxation parameters” (*s*) that effectively smooth the constraint. We therefore replace the constraints in Eqs 7, 8, and 10 with

$$\left(l_{i\text{max}}^{\prime }-l_{ijk}^{\prime }\right)F_{ijk}^{\prime }+s_{aijk}\geq 0,$$ (22)

$$F_{ijT}^{\prime }\;\int_{0}^{t}\;\;\;F_{ijL}^{\prime }dt+s_{bij}=0,$$ (23)

$$\begin{array}{c} \begin{array}{c} r_{F}^{\prime }\times F_{Fjk}^{\prime }+s_{cFjk}\geq 0,\\ -r_{H}^{\prime }\times F_{Hjk}^{\prime }+s_{cHjk}\geq 0, \end{array} \end{array}$$ (24)

with {*s*_*aijk*_, *s*_*bij*_ *s*_*cijk*_} ≥ 0. These relaxation parameters decrease the accuracy of the solution (compared to the solution of the non-smoothed problem), but by iteratively reducing their magnitudes, we can get arbitrarily close to the true solution [34].

We can further ratchet down the relaxation parameters by incorporating them into the cost function [35]. We make the smoothing terms decision variables (controls), and augment the cost function with the penalty

$$\begin{array}{c} \tilde{J}=J+\int_{0}^{T}\;\sum_{i,j,k}\left(c_{3a}s_{aijk}\left(t\right)\right)+\sum_{i,j}\left(c_{3b}s_{bij}\left(t\right)\right)+\sum_{i,j,k}\left(c_{3c}s_{cijk}\left(t\right)\right)dt \end{array}$$ (25)

where *c*_3*a*_, *c*_3*b*_ and *c*_3*c*_ are constants chosen to be sufficiently large [34]. Then, the optimizer tends to lower the magnitudes of *s*_*a*,*b*,*c*_(*t*), making the solution more accurate.

We started by not enforcing complementarity conditions (*c*_3*a*,*b*,*c*_ were 0 and *s*_*a*,*b*,*c*_(*t*) were allowed to take on any positive value) and then to increase the magnitude of coefficients *c*_3*a*,*b*,*c*_ as we increased the resolution on each mesh iteration. An exception to this was in the slack variable complementarity condition itself (21), which, on every mesh iteration except the first, was not enforced except through the augmentation

$$\begin{array}{c} \tilde{J}=J+\int_{0}^{⊤}\;\sum_{i,j,k}c_{2}p_{ijk}\left(t\right)q_{ijk}\left(t\right)dt, \end{array}$$ (26)

where *c*_2_ = 1 E-3.

The resulting cost function for the optimization (prior to normalization), was

$$\begin{array}{c} {\tilde{J}}_{\text{tot}}=\frac{1}{mgD}\int_{0}^{⊤}\;\sum_{i,j,k}(p_{ijk}+q_{ijk}+c_{1}{\dot{F}}_{ijk}^{2}+c_{2}p_{ijk}q_{ijk}+c_{3a}s_{aijk}+c_{3c}s_{cijk})+\sum_{i,j}c_{3b}s_{bij}dt \end{array}$$ (27)

The final issue, poor scaling, was partially helped by normalization, and further ameliorated through the automatic scaling feature (‘automatic-hybridUpdate’) in GPOPS-II [36]. Automatic scaling requires all unbounded variables to be redefined with finite bounds. These bounds are shown in S1 Table, and were chosen to be much larger than the expected magnitude of the variables, but small enough to improve scaling of the optimal control problem (see S1 Appendix for further details).

While the characteristic length for the computational problem was *l*_*B*_, we will report non-dimensional speed as the square root of the Froude number using the hindlimb maximum length,

$$\begin{array}{c} U_{H}^{\prime }=U/\sqrt{gl_{H\text{max}}}, \end{array}$$ (28)

following a common convention in other literature [1, 37].

We determined a best-fit normalized force-rate penalty constant ($c_{1D}^{\prime }$) for the Dalmatian documented in [37] moving with time constant ${\hat{T}}_{D}$. For all other cases, $c_{1}^{\prime }$ (dependent on $\hat{T}$) was scaled relative to $c_{1D}^{\prime }$ as $c_{1}^{\prime }\left(\hat{T}\right)=c_{1D}^{\prime }\frac{{\hat{T}}_{D}}{\hat{T}}$. The coefficients *c*_2_ and *c*_3*a*,*b*,*c*_ were not scaled.

#### Implementation of the trajectory optimization problem

The trajectory optimization problem was solved using a direct collocation method with *hp*-Adaptive Gaussian Quadrature, implemented in GPOPS-II (v. 2.1) [36]. The resulting nonlinear problem was solved using SNOPT (v. 7.4-1.1) [38, 39]. GPOPS-II mesh tolerance was set to 10^−4^.

To seed the trajectory optimization problem, values at each of the 16 initial grid points for each variable were chosen from a uniform pseudo-random distribution within the bounds of each variable. An exception is the horizontal position state variable, which was set as [0, *D*′] for the start and ending conditions, respectively, and interpolated linearly between those values for intermediate grid points.

The algorithm for finding the pseudo-global optimum (the optimum among all valid solutions) is as follows. An initial randomized guess was passed to GPOPS-II with one mesh refinement step. For this initial step, Eq 21 was explicitly enforced with *c*_2_ and all *c*_3*a*,*b*,*c*_ set to 0. This step allowed the program to more quickly approach a feasible region. The output was then down-sampled and passed back to GPOPS-II, this time with two mesh iterations steps, no explicit enforcement of Eq 21 and with the fully augmented objective (Eq 27), with *c*_2_ = 1 E-3 and *c*_3*a*,*b*,*c*_ = [100 10 10] (relaxation parameter penalty coefficients for Eqs 22, 23 and 24, respectively). This process was repeated two more times, with three and finally eight mesh iterations, these times setting $c_{2}=1\;\text{E}\;\text{-3}\;$ and *c*_3*a*,*b*,*c*_ = [1000 100 100]. The final solution was saved and the problem reset with a new random guess, until the required number of random guesses were explored. Other settings used for SNOPT and GPOPS-II are listed in S2 Table.

For exploring the effect of $c_{1D}^{\prime }$ on the solution in the Dalmation test case, 100 initial guesses were used. For the three detailed test cases, 500 initial guesses were used. For comparison to the large empirical dataset from [40], the number of guesses depended on the speed, since lower speeds required longer solution times per guess but the final solutions were not strongly affected by the initial guess. For $U_{H}^{\prime }\leq 0.6$, 250 guesses were used; for $0.6<U_{H}^{\prime }\leq 1$, 500 guesses were used; for $1<U_{H}^{\prime }\leq 2$, 750 guesses were used; and for $U_{H}^{\prime }>2$, 1000 initial guesses were used.

Each solution was determined as “valid” on the basis of (1) satisfactory mesh error (lower than the tolerance of 1 E-4) and (2) satisfactory (low) constraint violation on grid points. The second criteria was especially critical for complementarity conditions, which were not explicitly enforced and were violated more often than the other constraints.

Among valid solutions, the lowest cost solution was selected as the (pseudo-)global optimum as determined by *J*_tot_ (Eq 19). That is, since constraints were checked through the above validation step, the augmented parts of the objective (regarding constraint violation) were ignored to determine energetic optimality of one solution as compared to the others.

### Empirical data

The model requires five inputs: stride length, speed, body length, center of mass position (determined by $m_{F}^{\prime }$), and limb lengths. As validation of the model, duty factor, phase offset (fraction of stride between left-hind contact and contact of a given limb), and ground reaction force profiles are also required. These empirical data were acquired for single speeds from three studies [37, 41, 42], representing dogs moving at a slow walk, medium walk, and trot, respectively. In addition, [40] provides curves for duty factor, stride length, and phase offset as a function of speed from a large dataset. While all these studies supply a length calibration (*e.g*. shoulder height, withers height), they do not supply all necessary body proportions (except for [37], which supplies shoulder and hip locations in Fig 12 of that study). These additional proportions were taken from sagittal view images of the breed standard from the American Kennel Club website [43–45]. The acquired parameters are summarized in Table 2.

**Table 2:** Empirical data used for validation and as input (bold) for the model.

Main Source	[41]	[37]	[42]	[40]
Breed	Mix: 50% Rhodesian Ridgeback	Dalmation	Labrador Retriever	Belgian Malinois
$m_{F}^{\prime }$	**0.63**	**0.61**	**0.66**	**0.63** *
$l_{H}^{\prime },l_{F}^{\prime }$	**0.91, 0.75**	**0.89, 0.79**	**0.86, 0.72**	**1.01, 0.92**
*l*_*B*_ (m)	0.63	0.61	0.52	0.48
Source for proportions	[45]	Fig 12 in [37]	[44]	[43]
$U_{H}^{\prime },D^{\prime }$	**0.34, 1.3**	**0.39, 1.21**	**1.15, 2.0**	$D^{\prime }=1.04+1.36U_{H}^{\prime }$
Gait classification	Walk in lateral couplets lateral sequence	Walk in singlefoot lateral sequence	Running trot	Variable
PL	0.15	0.20	0.50	$U_{H}^{\prime }$ dependent
DFh	0.65	0.61	0.40	$U_{H}^{\prime }$ dependent
DFf	0.69	0.67	0.51	$U_{H}^{\prime }$ dependent

Symmetrical gait classification according to Hildebrand [9]. DFh and DFf are mean duty factor of the hind and forelimbs, respectively.

*Not reported at [40]; derived from [41]

Forelimb length (*l*_*F*max_) was taken as the length from manus to shoulder joint in standing. Hindlimb length (*l*_*H*max_) was taken as length from pes to hip joint in standing. Body length (*l*_*B*_) was taken as hip to shoulder joint in standing. Masses were assumed concentrated at shoulder and hip, following [41]. To determine center of mass location (or, equivalently, the anterio-posterior body mass distribution [46]), a balance of torque approach was used following [47]. Since the angular acceleration about the center of mass must be zero during steady, periodic locomotion, the net torque produced by the hindlimbs in the sagittal plane must be equal and opposite to the forelimbs. Assuming the trunk is approximately horizontal during the stride, then

$$m_{F}=\frac{2}{gT}\int_{0}^{T}\;\hat{y}\cdot F_{\text{Fm}}\left(t\right)dt\;\;\;\;\;\text{and}$$ (29)

$$m_{H}=\frac{2}{gT}\int_{0}^{T}\;\hat{y}\cdot F_{\text{Hm}}\left(t\right)dt.$$ (30)

where *m*_*F*_ and *m*_*H*_ are the masses supported by the fore and hindquarters, respectively, $\hat{y}$ is the vertical unit vector, and **F**_Fm_ and **F**_Hm_ are the empirical mean fore and hindlimb ground reaction force vectors. Note that we defined *m* = *m*_*H*_ + *m*_*F*_, which may deviate slightly from the reported masses in the associated studies due to measurement error.

Forces and footfall sequence were derived from Figs 8 and 9 in [41], respectively. Ground reaction forces and footfall sequence were also pulled from Fig 5a in [37], Fig 1 in [42], and Fig 3 in [48], for comparison to modelled predictions.

The standard error in mean phase offset from [41] was found by the authors to be small, and so was not reported for most limbs. Variation in footfalls could not be calculated from [37] as these authors reported only one footfall sequence at the speed of interest. The standard deviation in duty factor was reported by [42] in their Table 1, but error in phase offset was not reported. For this, we took the range of phase offsets observed in Fig 1 in [42] as a measure of the natural variation in phase offset. We used the same approach for both phase offset and duty factor in [48], using their Fig 3b. Therefore, in the present paper, error bars in gait diagrams represent twice standard deviation where available, and range of observed values otherwise.

We required empirical gait transition speeds to compare to our modelling results. The concept of “a transition speed” in dogs is somewhat misleading, and Maes *et al*. [40] observed something better described as a *transition zone*: speeds where dogs were almost equally likely to be utilizing one gait or another. We quantified the probability of dogs choosing one gait or another using their data (Fig 4 in [40]), and binned observations in $U_{H}^{\prime }=0.1$ increments. Trotting and Pacing were combined as one gait, as were Transverse, Slow Rotary and Fast Rotary Gallops. The relative frequency (number of observations for a particular gait divided by the total number of observations at that speed) are shown in S1 Fig. We defined the gait transition point as the speed where the relative frequency of one gait (*e.g*. walking) transitions from greater than 0.5 to less than 0.5.

#### Gait terminology

For all simulation results, left-hind touchdown (LHTD) is set at *t* = 0, and phase offsets are timed relative to LHTD. For ease of comparison (unless otherwise stated), optimal gaits are transformed so that, if left fore touchdown occurs after right fore touchdown, the left and right forelimbs are swapped. For trotting and galloping speeds, the opposite transformation is used. Since the model is planar, these phase transformations result in no change in cost while making all two-beat gaits “trots” and all four-beat symmetrical gaits lateral sequence [9].

Another useful set of terms is the fraction of time between foot touchdown of left-hind and right-hind (Hind Lag), left-hind and left-front (Pair Lag) and left-front and right-front (Fore Lag). While these phase lags generally follow the basic terminology used by Abourachid *et al*. [49], our Pair Lag (PL) differs from their definition (PL_Abourachid_ = 1 − PL for symmetrical gaits), since we use the left-hind leg as reference while Abourachid *et al*. used a front limb.

Symmetrical gaits occur when the time between contact of the left and right hindlimbs and left and right forelimbs are about half of the stride cycle (HL ≈ FL ≈ 0.5). For these symmetrical gaits, the pair lag fully parameterizes the footfall sequence. If phase offsets are transformed as described above, we have only 0 ≤ PL ≤ 0.5 in walking, and 0.5 ≤ PL ≤ 1 for running.

We follow the symmetrical gait terminology of Hildebrand [9]. Lateral couplets indicates 1/16 < PL ≤ 3/16, singlefoot is 3/16 < PL ≤ 5/16 and diagonal couplets is 5/16 < PL ≤ 7/16. Following transformation, all symmetrical four-beat gaits have 1/16 < PL ≤ 7/16. Two beat symmetrical gaits are the pace (PL ≤ 1/16 and 15/16 < PL) and the trot (7/16 < PL ≤ 9/16). These conditions and others are shown graphically in S2 Appendix. This appendix also presents a detailed comparison of our symmetrical solutions (without phase transformation) to Hildebrand’s extensive dataset [50].

## Results and discussion

### Initial validation and fitting of rate cost

The simulation was run for the morphology, speed and stride length derived from [37], as outlined in Table 2 for a number of rate penalties (*c*_1_ in Eq 18). Empirically, the prescribed speed corresponded to a moderate walk [9]. The simulation spontaneously discovered a symmetrical singlefoot walk as cost-minimizing regardless of rate penalty, with pair lags of approximately 0.25 (close to the empirical value of 0.20).

**Fig 3:**
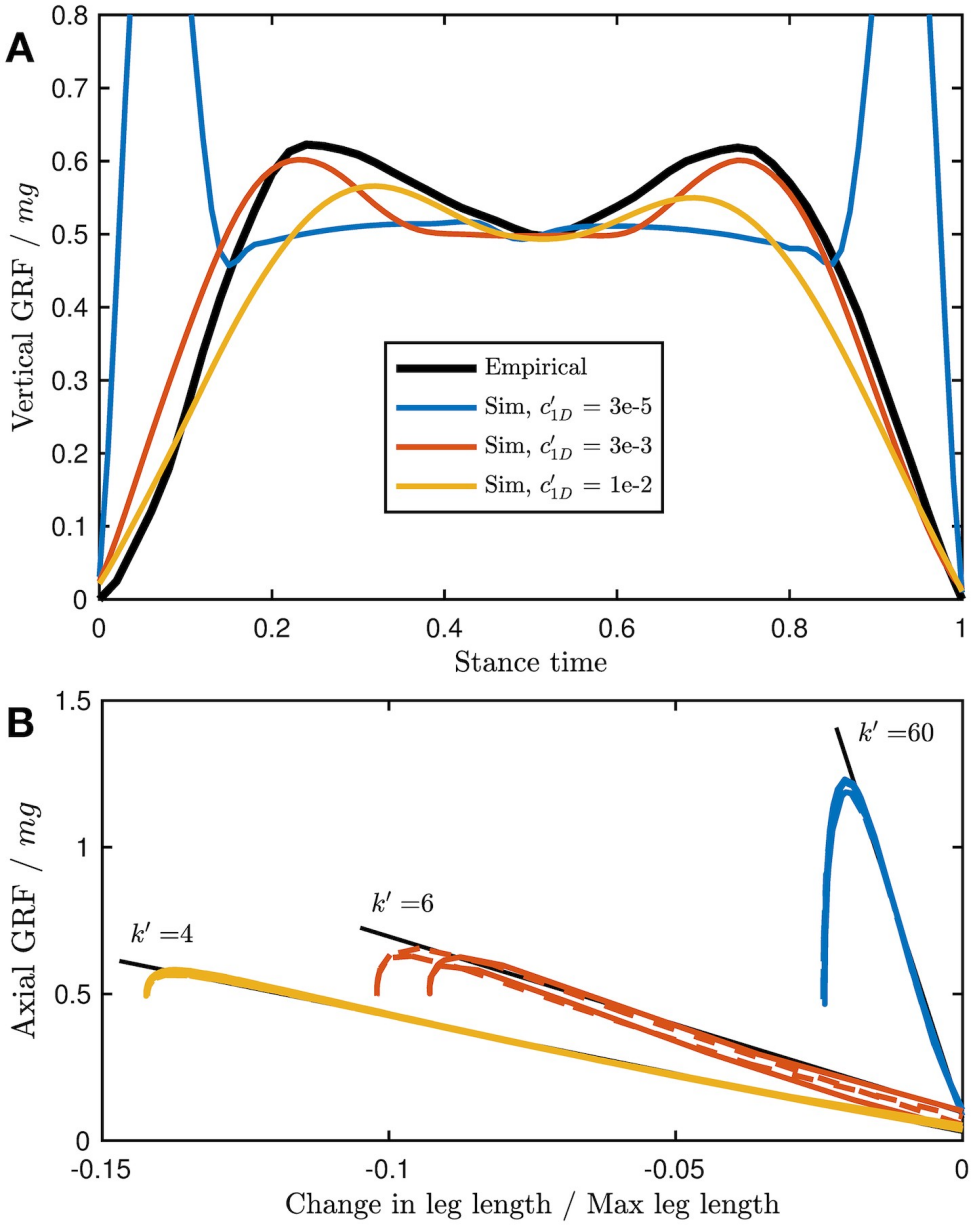
Empirical forelimb GRF (black) compared to model predictions for a range of force-rate penalty coefficients ($c_{1D}^{\prime }$). (A) At lower $c_{1D}^{\prime }$, the solution becomes more impulsive, as expected from a work-minimizing “bang-coast-bang” solution [23]. As $c_{1D}^{\prime }$ increases, the force peaks become more shallow, but in all cases the optimal solution maintains the double-hump profile characteristic of walking bipeds and quadrupeds under most situations. (B) The actuation is seemingly elastic, as indicated by the negative linear relationship between force and leg length change, for small changes in leg length from resting. However, there are no springs in the model. The “pseudo-elastic” actuation occurs because of the compromise between work and force rate. High force rate penalties lead to only small deviations from pseudo-elastic actuation and low apparent stiffness (*k*′), and small force rate penalties deviate substantially from pseudo-elasticity with high apparent stiffness. The “hook” in the force-length curve occurs when the leg shifts to stance, modulating the force to ∼0.5 *mg*. Morphological data for the simulations, and empirical ground reaction forces, are derived from [37] for a Dalmation (see also Fig 5B).

For all rate penalties, the simulation predicts a “double-hump” shape in ground reaction force to be optimal (Fig 3A), a common characteristic of both bipedal and quadrupedal walking [51]. The amplitudes of the peaks are affected by the magnitude of the force-rate cost. A purely work-based objective is expected to have impulsive foot-down and push-off [22, 23, 30, 52], but this would incur a large force rate penalty. The model compromises on a solution with smooth GRF, with larger peaks if the force rate penalty is small, and smaller peaks if the penalty is large.

Despite the model having no springs, the solutions exhibit spring-like actuation (Fig 3B)– but the extent depends strongly on the force-rate penalty. For all $c_{1D}^{\prime }$, the axial ground reaction forces vary linearly with changes in leg length, for leg lengths near to resting. This property gives each case an apparent stiffness that increases as the force rate penalty decreases. However, for small force rate penalties, the force to leg-length relationship deviates substantially from linearity the farther leg length is from resting. For higher force rate penalties, the spring-like behaviour is maintained throughout the entire compression-extension cycle.

**Fig 4:**
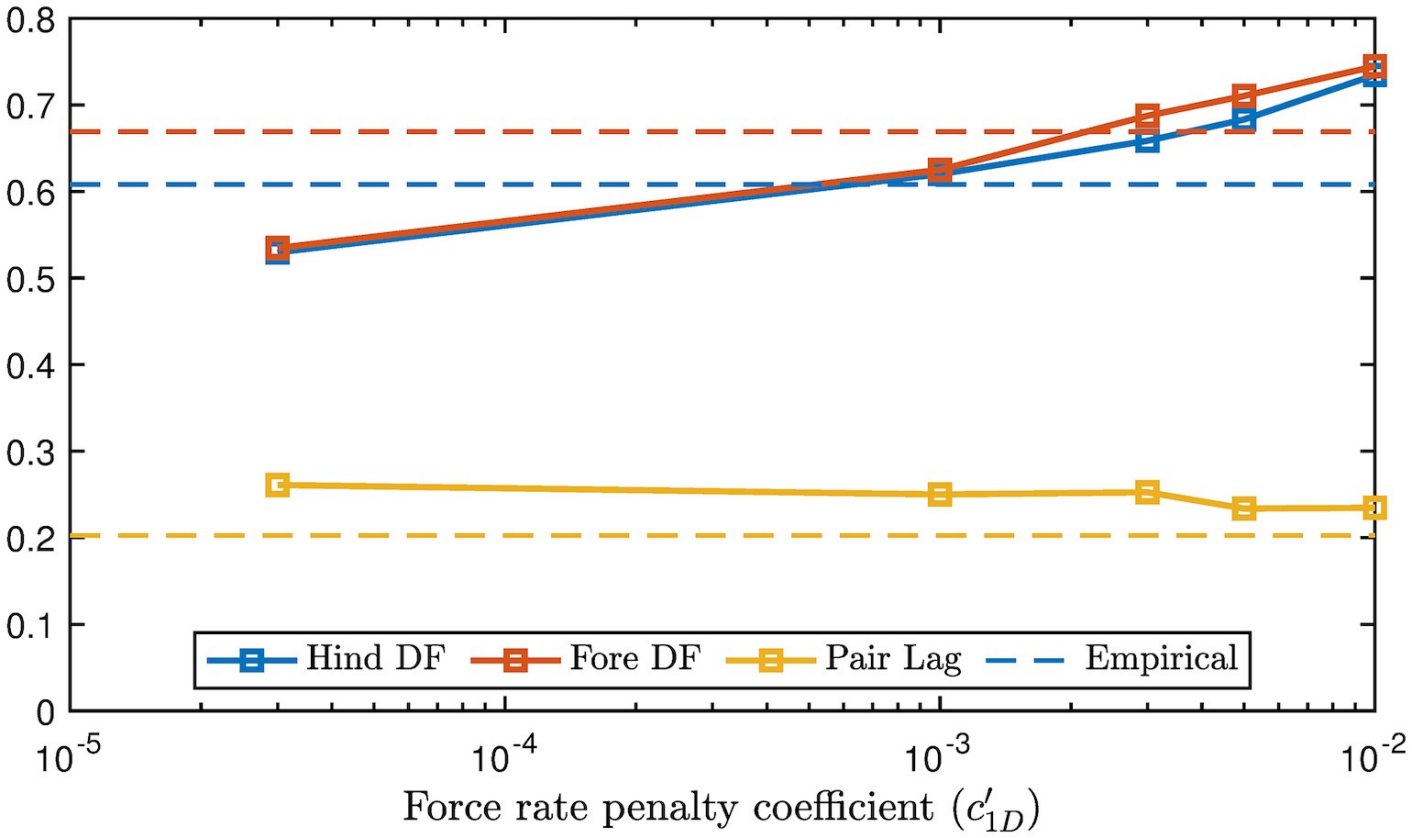
Optimal duty factors and pair lag *vs* magnitude of force rate cost coefficient ($c_{1D}^{\prime }$) at $U_{H}^{\prime }=0.39$. As force-rate penalties increase, duty factor also increases. Optimal duty factors in fore and hind remain close for all $c_{1D}^{\prime }$. Pair lag decreases slowly with force rate penalty. At no point do optimal duty factors and pair lags simultaneously match the empirical values (dotted lines) from [37]. The morphological data is based on a Dalmation from [37] (see also Fig 5B).

Five multipliers on rate penalties are compared in Fig 4. As rate penalty increases, both fore and hindlimb duty factors increase and remain relatively similar. Duty factor is strongly dependent on rate penalty because mean vertical force must remain constant over a *stride* (supporting weight), but a longer actuation time allows the mean vertical force in *stance* to be lower. At the lowest rate penalties, duty factor and pair lag approach the work minimizing solutions of 0.5 and ≈ 0.25, respectively, for symmetrical walking [53]. Optimal pair lag is fairly insensitive to rate penalty, but exhibits a slight decrease with increased force-rate cost.

While either fore or hind duty factor can be tuned to be arbitrarily close to empirical values, matching both is not possible through adjusting rate penalty alone in this case. Furthermore, since phase offset is relatively insensitive to rate penalties, it is likewise difficult to tune. The results of this analysis imply that, while adding a rate penalty enhances agreement with empirical data, it alone cannot explain the discrepancy between work-minimizing solutions and empirical results, at least for this highly simplified model. This finding is in contrast to human locomotion models, where the simple addition of a force-rate penalty enhances empirical agreement substantially [28, 30].

The cost of the force rate penalty relative to cost of transport increased from 12%, to 24% and finally 33% at $c_{1D}^{\prime }=3\;\text{E}\;-5\;,\;3\;\text{E}\;-3\;\text{and}\;1\;\text{E}\;-2\;$, respectively. $c_{1D}^{\prime }=3\;\text{E}\;\text{-3}\;$ matches the empirical GRF profiles (Fig 3) and duty factors (Fig 4) for this test case fairly well. It is used as the coefficient of choice for further exploration in the remaining test cases.

### Detailed comparison to four test cases

**Fig 5:**
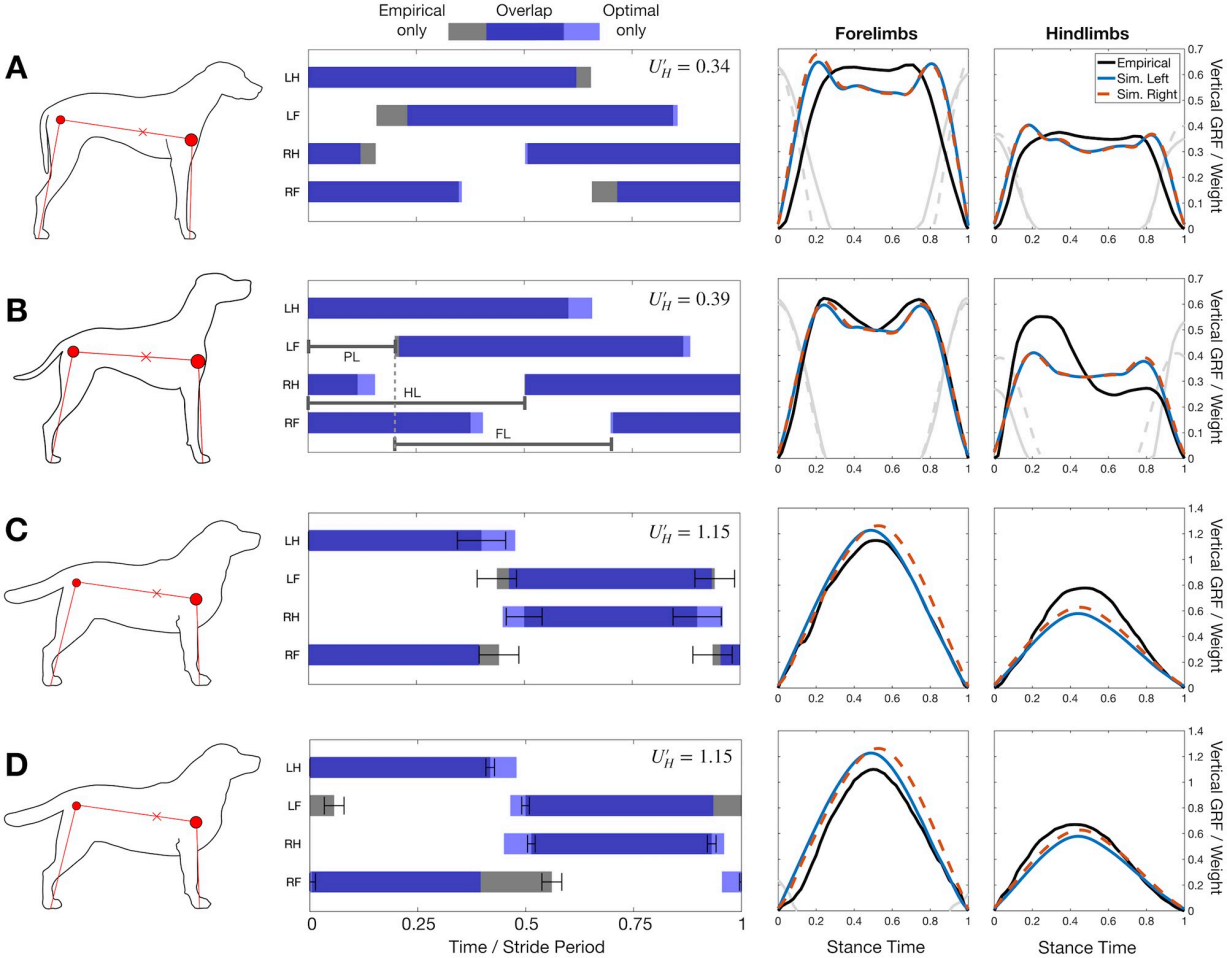
Optimal solutions compared to empirical data for four test cases. Left column shows an outline of the dog breed in question, with the approximate points used as morphological measurements. Middle column shows that gait diagrams for the empirical (grey) and optimal solutions (light blue) have substantial overlap (dark blue). Right two columns demonstrate that empirical ground reaction forces (GRF, black lines) qualitatively match optimization results. The optimization GRF from left (solid blue line) and right limbs (dashed red line) are very similar, reflecting the symmetrical solutions that are discovered. GRFs from double support are shown as grey lines (solid for empirical, dotted for simulation). At slow speeds (A-B), the simulation discovers a singlefoot walking gait, while at an intermediate speeds (C-D) it discovers trotting, matching natural gait choice in dogs. All optimizations use $c_{1D}^{\prime }=3\;\text{E}\;\text{-3}\;$. Contours adapted with modification from (A) [55], (B) [56], and (C-D) [57]. Empirical data and morphological measurements from [41, 45] (A), [37] (B), [42, 44] (C) and [44, 48] (D). For left column, circles show shoulder and hip positions, with relative size indicating the relative mass carried at these points. “x” marker indicates centre of mass position. For middle column, LH = Left Hindlimb, RH = Right Hind, LF = Left Fore, RF = Right Fore. Error bars in gait diagrams are described in the methods.

Four cases are examined in detail in Fig 5. In the first two cases, the optimization correctly predicts a symmetrical, four-beat walking solution with double-hump ground reaction forces. In the third and fourth case, the optimization correctly predicts a two-beat run with near-simultaneous fore-hind contact and single-hump GRFs. The optimization also correctly predicts the gait to be a “grounded run” [54], in the sense that it exhibits in-phase potential and kinetic energy changes, but no flight phase. For trotting, two empirical results from related studies [42, 48] are compared to the same optimization solution, demonstrating the range of normal behaviour in trotting for this breed. Overall, the simulation matches either empirical result fairly well, demonstrated by the substantial overlap of stance and similar shape of the GRFs.

The substantial overlap between optimization and empirical gait diagrams demonstrates the success of the optimization approach. It must be emphasized that any of the 5040 quadrupedal foot contact sequences could have been selected by the optimization. While, in a planar sense, many of these gaits are the same (for example, there’s no distinction between a lateral and diagonal sequence gait), there are still 2352 event sequences that are unique in a planar sense. Moreover, the model could even exclude limbs, adding more options to its repertoire.

**Fig 6:**
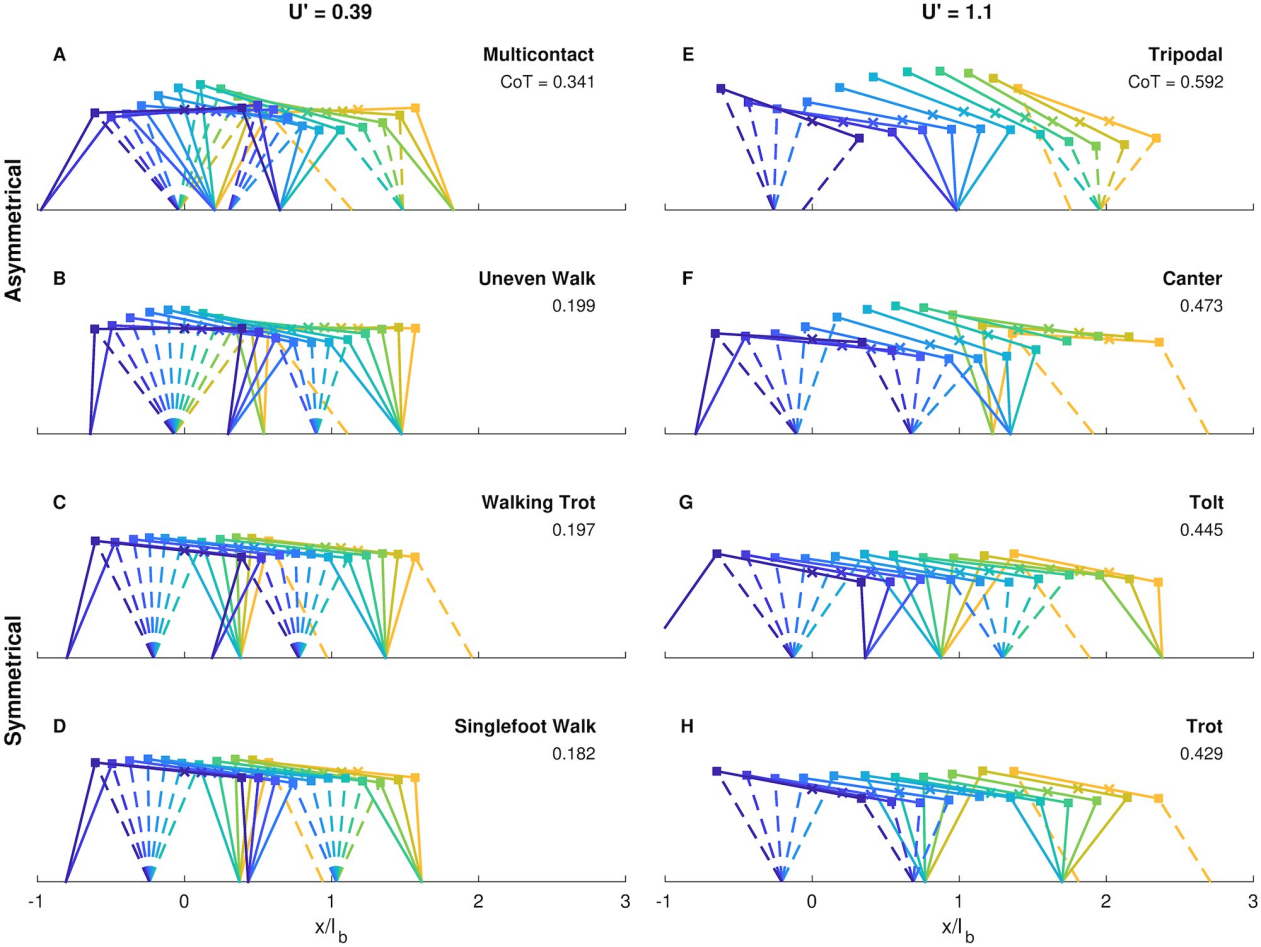
These locally optimal solutions are a sample of the diverse gaits possible with the model. Each stick figure is a snapshot from a different part of the gait cycle. The first still (leftmost, purple) is at touchdown of the left hindlimb. Each still is separated from the next in time by one tenth of the stride time, culminating at the next left-hind touchdown. The “x” marker represents center of mass location. At both a walking speed of $U_{H}^{\prime }=0.39$ (A-D) and running speed of $U_{H}^{\prime }=1.1$ (E-H), asymmetric (A,B,E,F) and symmetric gaits (C,D,G,H) are possible. *y*-axis and *x*-axis scales are equal.

Fig 6 shows some examples of locally optimal gaits discovered by the optimizer. These included asymmetrical (Fig 6A, 6B, 6E and 6F) and symmetrical gaits (Fig 6C, 6D, 6G and 6H), some that look like patterns utilized by animals (Fig 6C, 6D, 6F–6H), and some that are not typical for quadrupeds (Fig 6A and 6B). The model also sometimes used fewer than four limbs (Fig 6E). Yet the pseudo-globally optimal gaits are readily identified as those utilized by dogs at those speeds (Fig 6D and 6H) and match the empirical solutions fairly closely (Fig 5A–5D).

Only the second case (Fig 5B) was “tuned” with the force rate penalty; the same penalty was used in all other cases. Yet even within this tuning, the optimization always selected a gait that was qualitatively similar to the empirical data (Figs 3 and 4). These results come despite fairly rough empirical estimates, varying morphological parameters, and many simplifying assumptions in the model (avoiding springs, most joints, and using the simplest possible mass distribution).

Forces are predicted fairly well, at least qualitatively in terms of shape and magnitude. While shapes of the ground reaction forces are an optimization decision, the relative magnitude of these forces are highly constrained. The mass distributions used were taken from empirical vertical forces in the first place (Eqs 29 and 30), and kinematic periodicity ensures that net torque around the body is zero. Furthermore, mean net vertical force must equal body weight. Assuming the body remains relatively horizontal, then these two conditions completely determine the mean vertical force produced by either pair of limbs. While these arguments emphasize that little merit should be seen in matching relative magnitudes (apart from duty factor predictions), they do show that the quadruped’s center of mass must be biased towards one pair of limbs to achieve fore-hind differences in (mean) GRF magnitude. Without a bias in COM position, there can be no difference in mean vertical forces produced by the fore and hindlimbs; single-point mass models of quadrupedal locomotion cannot capture this effect [58].

While the mean magnitude of GRF is not very informative, the shape is. The optimization predicts a double-hump GRF profile for walking (Fig 5A and 5B), and single hump for trotting (Fig 5C and 5D). These predictions are born out in the empirical cases, although the magnitude of the force peaks in the slowest case is substantially lower than predicted. In the intermediate walking case (Fig 5B), the empirical hind GRF trace was derived from simultaneous force-plate contact of fore and hindlimbs using a questionable method [37], and is likely skewed for that reason.

In bipedal locomotion, the double-hump shape arises from the pre-footstrike pushoff [59–61], a dynamic “trick” for minimizing energetic losses at foot contact. The pre-footstrike pushoff redirects the center-of-mass velocity so that it is less in line with the leading limb about to touch down, thus reducing the amount of negative work the leading limb needs to do [20, 28]. The same “trick” seems to be discovered by the model; a second peak in the trailing limb ground reaction force occurs prior to the first peak in the leading limb.

In trotting, the roughly symmetrical, single hump profile is representative of a short, largely vertical contact of the limbs, which are work-minimizing in running for point-mass bipedal models [20]. As with the walking solutions (Fig 3A), the impulsive work-minimizing solution is smoothed by the force rate penalty.

**Fig 7:**
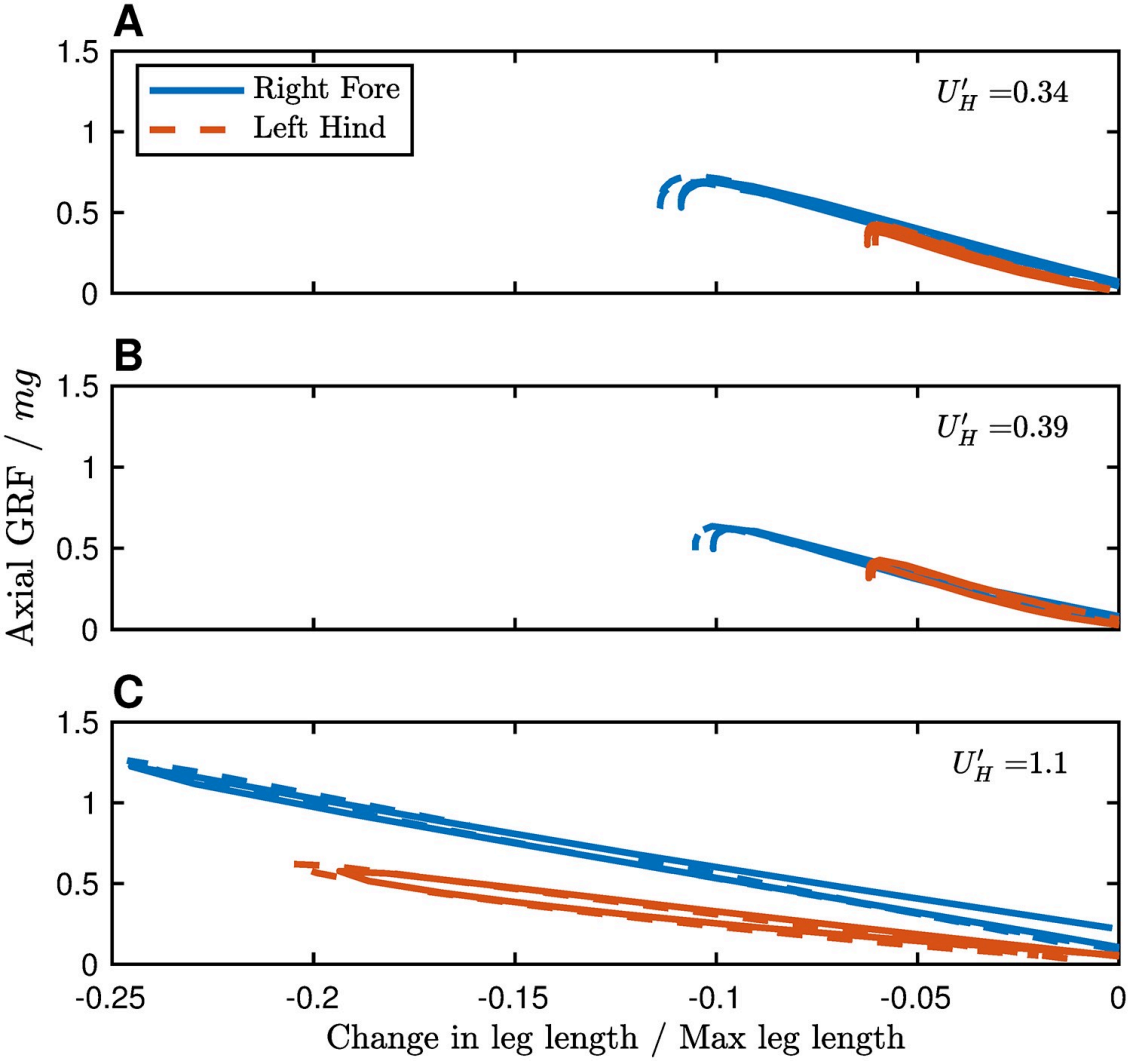
Actuation shows an elastic response to leg length change, despite a lack of elasticity in the model. At both walking speeds (A,B), the apparent stiffness appears similar between fore and hind legs (when normalized to maximum leg length). In trotting, however (C) there is a substantial difference in apparent stiffness between the fore and hind legs. Data are from the same simulations shown in Fig 5.

Fig 7 shows leg force plotted against leg length change from resting length (normalized to maximum leg length). All forces exhibit pseudo-elastic actuation, with more pronounced deviation from elastic at slower speeds. Spring-based models of locomotion replicate GRFs reasonably well [12, 13], but lack an energetic explanation for why spring-like actuation is chosen, or why leg stiffness should change with speed. In this inelastic model, pseudo-elastic actuation emerges as an optimal compromise between work and force-rate energetic penalties. There is no constraint that the same apparent stiffness must be used in all cases, however, or that actuation must be spring-like throughout the entire contraction-extension cycle.

Our interpretation contrasts with [31], who assert that all GRF shape characteristics (double hump, single humps) are from “oscillation modes” due to resonances from the spring-like actuation. These same features are present under inelastic work-based optimization (Figs 3 and 5, see also [22, 23, 30, 52]), and we contend that they are due to work minimization. Smooth, pseudo-elastic GRFs result from a trade-off between work and force-rate costs [29, 30], resulting in spring-like actuation.

While spring-mass systems are often used to approximate the emergent gait behaviour, they are not the only system that describes the behaviour well and should not be taken as a prerequisite for “spring-like” behaviour. Rather, spring-like behaviour emerges from energy minimization [20–22, 52], even in the absence of springs.

### Comparison to a large kinematic dataset

It is difficult to tell if differences between solutions in Fig 5 are due to differences in morphological input or prescribed speed. To control for these differences, the model was compared to a large dataset using Belgian Malinois dogs of similar sizes over a large speed range [40].

**Fig 8:**
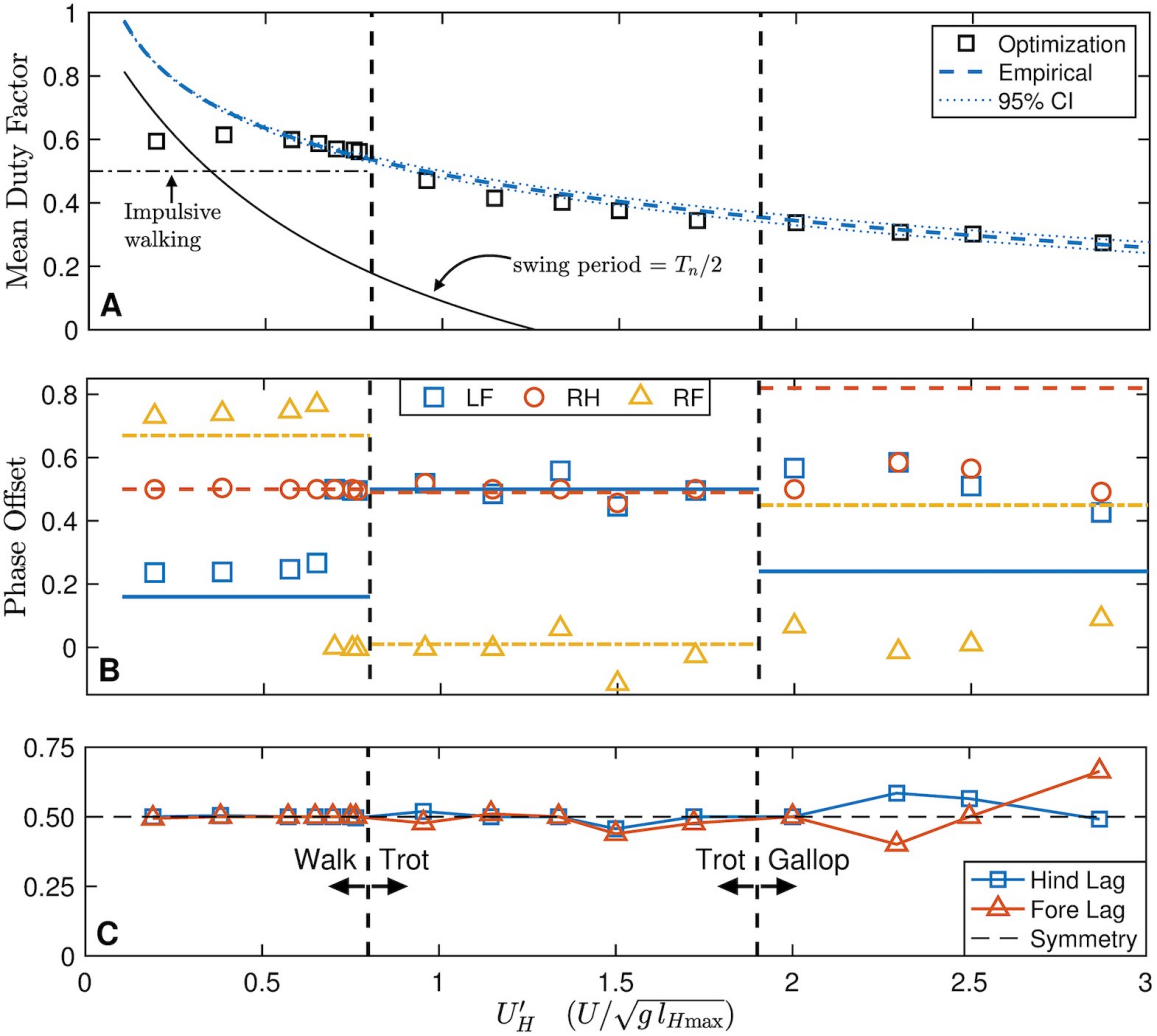
Pseudoglobal optimal solutions (markers) are compared to a large dataset for Belgian Malinois dogs. (A) A slowly decaying duty factor with increasing speed is predicted well by the model for medium to fast speeds, using $c_{1D}^{\prime }=3\;\text{E-}3$. At slow speeds, the model remains constant, slightly above impulsive predictions, while the empirical data approaches 1. A swing duration of half the natural pendular period (*T*_*n*_) of the leg (Eq 31) does not explain the discrepancy. 95% CI for empirical mean duty factor is shown as a thin dotted line. (B) The optimal phase offset matches empirical values well for walking and trotting speeds, and the optimal walk-trot transition is very close to the natural transition speed. However, the model does not transition to a gallop after the natural trot-gallop transition speed (C) Hind Lag (HL) and Fore Lag (FL) is shown against speed. A perfectly symmetrical gait would have HL = FL = 0.5; walking and trotting in dogs is highly symmetrical both naturally [50] and in the model. Galloping is an asymmetrical gait, and past the trot-gallop transition, the lowest-cost gait discovered by the model becomes asymmetrical. Empirical phase offsets are mean values for each gait. All empirical data from [40].

The results of the simulations are compared to empirical results in Fig 8. Optimal mean duty factor (averaged across all limbs) matches empirical values very well for $U_{H}^{\prime }\geq 0.5$, (Fig 8A) following its slow decay as speed increases. For $U_{H}^{\prime }<0.5$, the predicted duty factor is almost constant at approximately 0.6. As with the other test cases (Fig 5), the model predicts singlefoot walking as the optimal gait at low speeds, and trotting as optimal at high speeds (Fig 8B). Phase offsets are predicted well for all walking and trotting speeds, with all phase offsets lying within 0.1 of the empirical values for $U_{H}^{\prime }\leq 1.75$. The model predicts a sharp transition to a trotting gait between $U_{H}^{\prime }=0.65$ and 0.7, close to the empirical transition point of $U_{H}^{\prime }=0.8$ (S1 Fig). Curiously, the simulation chooses a transition speed almost exactly at the slowest self-selected trotting speed ($U_{H}^{\prime }=0.65$) observed by Maes *et al*. [40] (S1 Fig). While a true gallop never emerges from the optimization, even well past the natural trot-gallop transition of $U_{H}^{\prime }=1.9$ (S1 Fig), slight asymmetry begins to emerge from the solution for $U_{H}^{\prime }>2$ (Fig 8C).

#### Response of optimal duty factor to speed

For $U_{H}^{\prime }>0.5$, optimal values follow the empirical trendline closely (Fig 8A). For $U_{H}^{\prime }<0.5$, the optimal duty factor levels off at about 0.6, slightly above the work-minimizing prediction of 0.5 for a walk [53]. A similar pattern, where constant duty factor suddenly shifts to a decay with increasing speed, was also observed by Hubel and Usherwood [29] in their biped model with an analogous work + rate penalty objective (though their rate penalty was peak-power, rather than force rate).

Work-minimal walking should have DF = 0.5 because double stance involves costly simultaneous positive and negative work [23, 61]. Work-minimal running gaits should have DF = 0 because a vertical impulse at stance avoids costly fore-aft decelerations. The increase in duty factor above work-minimizing predictions in our model is due to the force rate penalty, which allows both these additional costs in order to avoid impulses. However, as speed increases, using a large stance duration involves ever-increasing excursion angles, and so larger and larger fore-aft forces, increasing work costs. Therefore, the optimal solution is to decrease duty factor as speed increases to manage this tradeoff.

It is interesting that the simple addition of a rate penalty yields such high agreement with duty factor at high speeds, especially since the model does not include massive limbs, springs, or a compliant back, nor does it use the correct (galloping) gait at high speeds. If rate penalties (whether force/time, power or activation penalties) are real phenomena, we would expect their influence to be most pronounced at higher speeds, where, since stride time is shorter, by necessity forces must be produced more quickly, power is higher, and muscles must be activated more frequently.

The discrepancy at lower speeds can be partially explained by leg swing dynamics. There is a cost to swinging legs during locomotion [62, 63] that is minimized when the leg is swung close to its natural frequency [64]. As a result, quadrupeds (including dogs) tend to decrease stance duration, rather than swing duration, as speed increases [40, 65].

If swing costs dominated, at all speeds it would be advantageous to the animal to adjust its duty factor such that swing time was exactly half the natural pendulum period of the leg, *i.e*.

$$\begin{array}{c} D\;\;F=1-\frac{T_{n}}{2T}, \end{array}$$ (31)

where *T*_*n*_ is the leg natural pendulum period and *T* is stride period. Taking *T* as given (based on empirical data), and using the allometric equations from [66] for the *T*_*n*_ of dog legs, we can generate the predicted curve shown in Fig 8A. It takes on a similar shape to the empirical data at low speeds, but lies well under the curve. According to the data from [40], dogs drive their legs in swing at about half the natural pendulum period on average during walking. The reasons for this are unclear; nevertheless, including limb moment of inertia might account for some of the discrepancy between the predicted duty factor and empirical data.

#### Response of phase offset to speed

Phase offset for each limb from the optimal solutions are compared to empirical data from [40] in Fig 8B. At low speeds, the model predicts a fore-hind phase difference (pair lag) between 0.23 and 0.27, close to the 0.25 phase predicted from impulsive walking with even mass distribution and equal fore and hindlimb lengths [53, 67]. The empirical pair lag for walking of 0.16 ± 0.05 (mean ± standard deviation) does not differ greatly from either of these values. For $0.7\leq U_{H}^{\prime }\leq 1.15$, the optimal solution is a symmetric trot, with closely-associated fore-hind contacts. Above $U_{H}^{\prime }=1.2$, the fore-hind contact becomes dissociated, with forelimbs leading or lagging associated hindlimbs by 0.05 to 0.11 *T*. For $U_{H}^{\prime }\geq 2.5$, the gait becomes somewhat asymmetrical, with either the fore or hind pair exceeding a 0.5 phase difference. In no case did a galloping gait emerge as globally optimal, despite the model settling on galloping as locally optimal in a number of cases. This finding agrees with others who have analyzed alternative rigid back models [31, 68].

The difference in cost between galloping and trotting is slight, however. For example, at $U_{H}^{\prime }=2.8$ the model spontaneously discovered a galloping solution with CoT = 0.58, only 3% higher cost than the optimal trotting solution at the same speed (CoT = 0.56). Previous studies have shown that galloping becomes optimal at high speeds with an elastic, compliant spine [69–72]. Future work could add active compliance to our model’s trunk, to see whether true elastic return is necessary for the optimality of galloping, or whether any form of compliance (even active compliance) makes galloping more economical.

Despite initializing the optimization from over 8000 randomized initial guesses, only four-beat walking and two-beat running (or slight deviations thereof) were discovered as pseudo-global optimal solutions. This is strong evidence (but not definitive proof) that these are globally optimal solutions for the quadrupedal configuration investigated (Fig 1) with a work-based cost and force-rate penalty.

The discovery of trotting (as opposed to tolting) as energetically optimal in running is consistent with an analysis by Usherwood [73]. A dimensionless pitch moment of inertia can be defined as [74]

$$\begin{array}{c} \hat{I}=\frac{4I}{ml_{B}^{2}}. \end{array}$$ (32)

To a first approximation, for $\hat{I}>1$, tolting is energetically optimal; otherwise trotting is [73]. In our case, $\hat{I}=0.93$, not far from a more realistic estimate of 0.84 for dogs [37, 75]. Since these dimensionless moments of inertia are less than 1, we expect the model (and dogs) to find trotting to be less expensive than a running walk.

However, some deviation from exact trotting– perfect symmetry and fore-hind offset of 0.5– was observed at higher speeds. Moreover, while a clear singlefoot contact pattern was observed for $U_{H}^{\prime }<0.65$, and a clear trotting pattern emerged for $0.65<U_{H}^{\prime }<1.2$, at higher speeds the footfall pattern was less consistent. Pair dissociation became more common, but was not always optimal (for example at $U_{H}^{\prime }=1.8\;\text{and}\;2.2$) and the optimal gait became asymmetric (but regularly increasing asymmetry was not observed in the pseudoglobally optimal solutions; Fig 8C).

#### Variability in local optima

**Fig 9:**
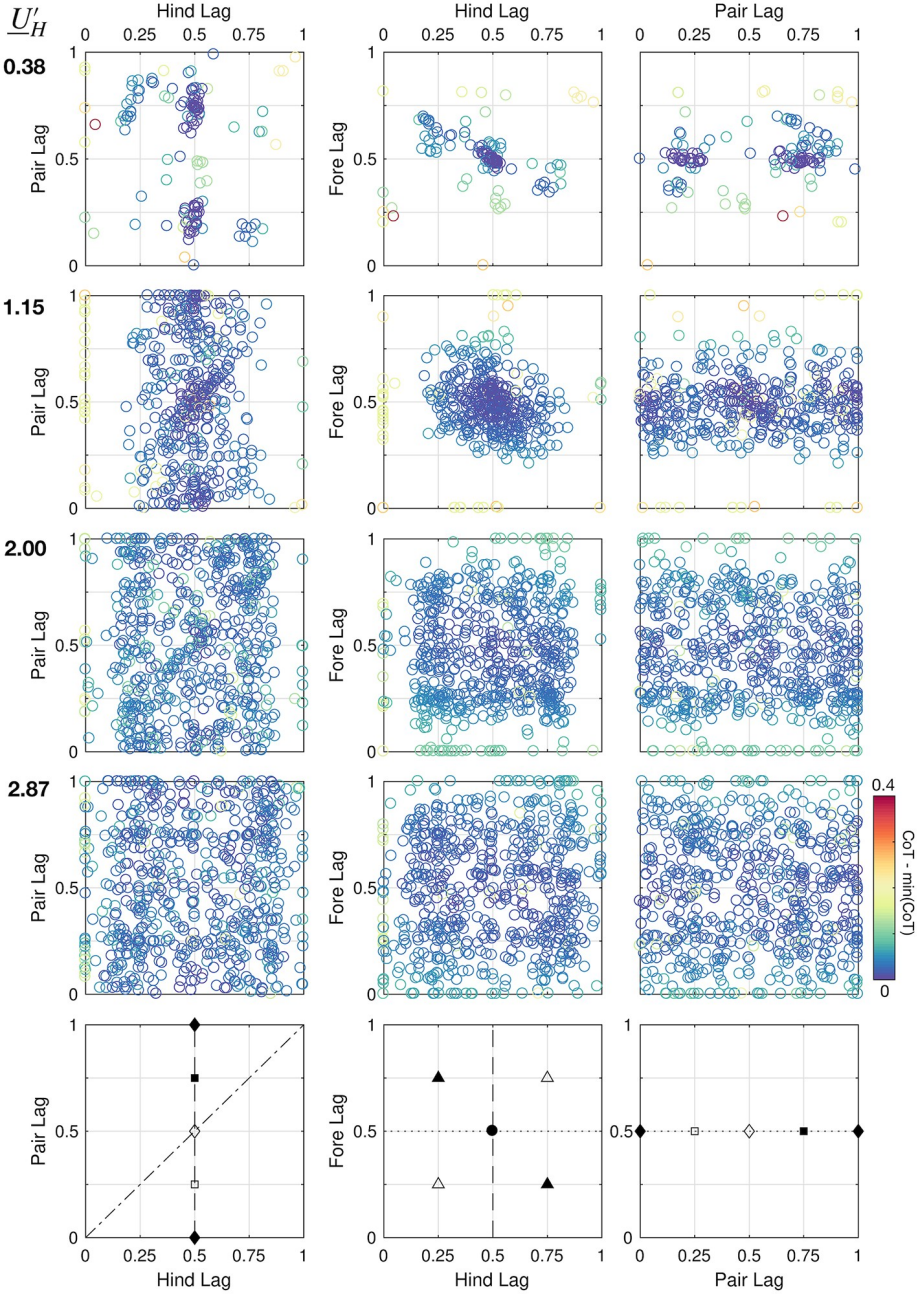
All local optima found for the model for each speed based on a Belgian Malinois morphology. Each row corresponds to a given speed condition, and each data point is a feasible local optimum arising from energy optimization from one random guess, with relative cost (CoT—min CoT for a given $U_{H}^{\prime }$) indicated by color. Solutions are plotted in terms of Hind Lag (HL), Pair Lag (PL) and Fore Lag (FL), defined in Fig 5B. As speed increases, solutions become more variable. Pseudo-global optimal solutions for each case are shown in Fig 8. Bottom row: Some recognizable gaits and their positions in the plots. Dashed line: hind symmetry; dotted line: fore symmetry; dot-dash line: simultaneous right-hind and left-front contact (as in a canter). Black square: diagonal singlefoot; white square: lateral singlefoot; black diamond: pace; white diamond: trot; black triangle: rotary gallop; white triangle: transverse gallop; black circle: perfect symmetry.

One reason for the lack of a consistent pattern may be that there are more variable locally optimal gaits at higher speeds. To explore this possibility, we plot phase offsets (without transformation) for all valid solutions for four speeds from the walking, trotting, slow gallop and fast gallop ranges (Fig 9). Each solution is both feasible (satisfying the constraints and dynamics), and locally optimal (that is, the NLP solver reported successful convergence). Each solution lives in a 3D space of foot contact phase lag: [Hind Lag, Pair Lag, Fore Lag] = [HL,PL,FL] (see Fig 5B and the “Gait Terminology” section). Color represents cost of each solution, with dark blue as low cost, yellow as high cost, and light blue as intermediate.

As speed increases, less solution clustering is observed. At a slow speed ($U_{H}^{\prime }\approx 0.4$), only two clusters emerge, at [0.5, 0.25, 0.5] and [0.5, 0.75, 0.5], representing symmetrical singlefoot walking (lateral or diagonal sequence, respectively [9]). At a higher speed ($U_{H}^{\prime }\approx 1.2$), clustering becomes less pronounced, but two clusters emerge; one at [0.5, 0.5, 0.5] (symmetrical trotting) and another at [0.5, 0, 0.5] (symmetrical pacing). However, unlike the walking case, symmetry is not as clearly optimal, as represented by the large scatter of solutions between 0.25 < {HL, FL} < 0.75 compared to $U_{H}^{\prime }=0.38$

At a slow galloping speed ($U_{H}^{\prime }=2.00$), clustering has all but disappeared. Still, bands of viable solutions emerge; in particular, many solutions emerge in the range 0.20 < {HL, FL} < 0.80 with any PL, while solutions are rare outside this range. A wide band of low-cost solutions emerges at FL = 0.5, with some bias towards HL = 0.5 (symmetrical trotting). However, small clusters also emerge at the nodes [{0.25, 0.75},PL,{0.25, 0.75}], representing galloping (transverse if HL = FL, rotary otherwise). Interestingly, solutions cluster around the PL = HL line in the leftmost plot, representing simultaneous contact of fore and hindlimbs. A canter would lie on this line.

Small clusters of high-cost solutions also emerge at [HL, FL] = [0, 1], representing a bound, half-bound, or pronk (depending on PL and the difference between HL and FL). These solutions appeared at all speeds, but were never globally optimal, reflecting the results of Xi *et al*. [31].

**Fig 10:**
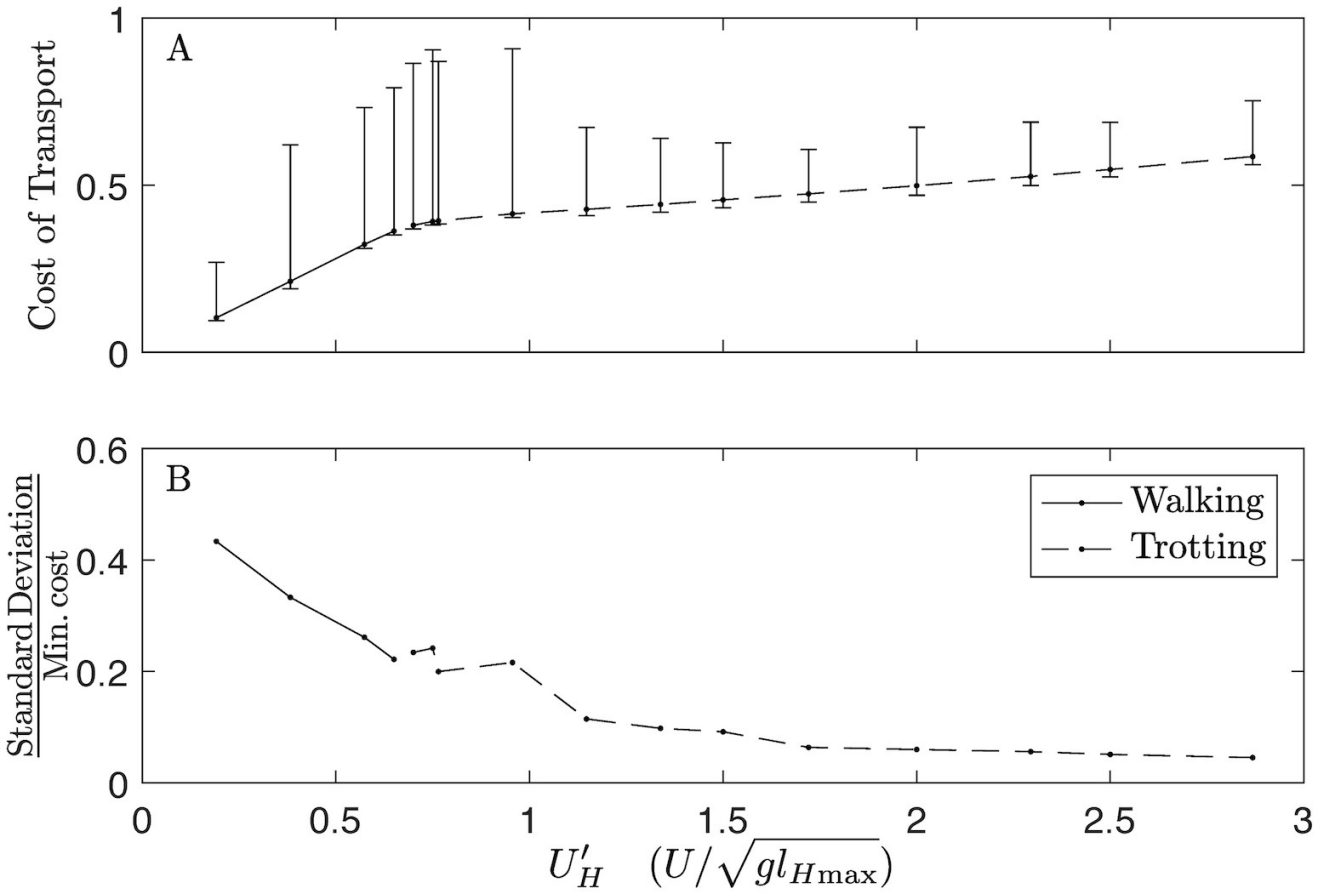
Changes in cost of transport with speed. (A) Median Cost of Transport increases linearly for walking speeds (solid line), but exhibits a sharp change to a slower rate of increase after the walk-trot transition (dashed line), mirroring the response to speed of walking and running observed in human data [77]. The range of the costs of feasible solutions (whiskers) increases up to the walk-trot transition, and then settles into a near-constant range. The distribution of costs is heavily skewed, as indicated by the median value approaching the minimum at all speeds. This indicates that the solvers tended to discover solutions with costs close to the minimal value, but occasionally were “trapped” in local optima with costs far from the minimum. (B) As speed increases, the standard deviation of the distribution of costs of transport gets smaller, relative to the minimal cost, indicating that the variance in costs of local optima is relatively smaller at higher speeds than at lower speeds.

Why are solutions more variable at higher speeds? One reason may be that phase and event sequence affect cost less at higher speeds than at lower speeds. Fig 10A shows how cost changes with increasing speed. Reflecting both human [76] and animal studies [14], the cost of transport in walking is highly sensitive to speed, while the cost of transport of running is much less so; however, minimum and median cost always increases with speed. Despite the increased costs in running, the range in CoT is fairly small at higher speeds compared to walking. Relative to the increases in cost, the CoT variability in feasible solutions decreases considerably from lowest speed to highest speed (Fig 10B) At high speeds, virtually all combination of phase are explored (Fig 9), with little change in cost (Fig 10). Optimizing locomotion at higher speeds appears to involve searching a flat and fuzzy cost landscape for the best gaits.

The variability in locally optimal solutions may somewhat reflect reality; dogs exhibit substantial natural variation in gait at higher speeds. For example, empirical data from [42, 48] indicate that duty factor and pair dissociation can vary at the same trotting speed in labradors (Fig 5C and 5D). The transition from trot-gallop is much less distinct than walk-trot (S1 Fig), and while trots and rotary gallops are the most common running gaits, dogs will also pace and use transverse gallops [40]. If the relative cost between these gaits are not substantial, then factors apart from energetics might have a greater influence on the locomotory strategy.

### Conclusions

We developed a minimally constrained quadrupedal model capable of any of the 2300+ planar contact sequences available to quadrupeds. Of those possibilities, it found two basic gaits– four-beat walking in singlefoot, and two-beat running trots or paces– as energetically optimal. It also transitioned spontaneously from walking to trotting at a realistic speed. This strongly suggests (but does not prove) that these gaits are globally optimal strategies at their respective speeds, at least for the modelling configuration used. Despite no enforcement of gait symmetry, the optimal gaits were highly symmetrical except at high speeds, as observed in natural gait.

The GRF profiles were smooth and double-hump in walking and single-hump in trotting. Double-hump walking and single hump running solutions emerge in work minimizing models, but are impulsive [22, 52]. The same shapes emerge in elastic models, but without an energetic penalty [12, 13]. In our inelastic model, the actuation *appeared* elastic despite no springs being present in the model. This “pseudo-elastic” activation occurs as an optimal tradeoff between work and force-rate penalties, potentially explaining why many animal gaits are well-described by spring-based models [11, 13], even when elastic recovery is minimal [19]. This interpretation complements the optimality of pseudo-elastic *collisions* in work-minimization [20], with pseudo-elastic *actuation* due to additional force-rate costs.

Though limb work was the chief contributor to cost, an additional force-rate penalty led to better predictions, especially with regards to duty factor. While the parameters affecting duty factor choice at slow walking remain a mystery, we believe that duty factor at higher speed is determined by the competing costs of work (lowering duty factor) and force rate (increasing it). However, little is known about the physiological mechanisms for this rate cost, and it could be an exciting avenue for further research.

Galloping did not emerge as optimal at high speeds in our model, but spring-based models exhibit the same result if elastic elements are not included in the trunk. Future work will examine whether galloping emerges as optimal if the torso is allowed to extend or contract actively. This would test whether elastic elements in the trunk are truly necessary for the optimality of galloping.

Despite simplified morphological parameters, the model predicted gait well at slow and intermediate speeds across several breeds of dog, demonstrating that detailed morphological modelling is not necessary to explain gait selection. Altogether, these results suggest that mammals are optimizing a work-based cost function with some form of force-rate related energetic penalty in steady locomotion.

## Supporting information

S1 Appendix Justification for variable bounds. (PDF)

S2 Appendix Comparing symmetrical solutions to Hildebrand’s results. (PDF)

S1 Table Optimization variable bounds. (PDF)

S2 Table Main settings for the optimization procedure. (PDF)

S1 Fig Relative frequency of walking, trotting/pacing and galloping as speed increases. Walking shown in blue, trotting/pacing in red and galloping in green. Observations normalized to total count in each bin. Data from Fig 4 in [40].
(TIF)

S1 File Optimal solutions for specific cases. Pseudo-globally optimal ground reaction forces, footfall positions and initial kinematic conditions are given for the results with varying force-rate penalty, as well as the other test cases, as shown in Figs 3–5.
(XLS)

S2 File Duty factor, phase and cost for all valid solutions discovered for a range of speeds using Belgian Malinois morphology, as displayed in Figs 8–10. ‘nlpinfo’ are the SNOPT exit conditions, with nlpinfo <10 indicating satisfactory convergence on a local optimum. Phase is relative to the left-hindlimb touchdown.
(XLS)
