# Supplementary material for: An inelastic quadrupedal model discovers four-beat walking, two-beat running, and pseudo-elastic actuation as energetically optimal
Source: PLoS Comput Biol. 2019 Nov 21;15(11):e1007444. doi: 10.1371/journal.pcbi.1007444 (PMC6871776; doi:10.1371/journal.pcbi.1007444)
Supplement: S2 Appendix — (PDF) [file pcbi.1007444.s002.pdf]

S2 Appendix: Comparing Symmetrical Solutions to Hildebrand's Results

The plots in Fig 9 represent a combination of symmetrical and asymmetrical solutions. Hildebrand [1] studied the symmetrical gaits of domestic dogs in great detail, and discovered that dogs (across a large range of speeds and breeds) choose a limited subset of symmetrical gaits from the total "gait space" available to them (every combination of duty factor and pair lag from 0 to 1). Does the limited scope of *Canis lupus familiaris* gait represent a fluke of evolution, a physical constraint, or is it the result of an energy optimization process?

In Fig 11 we plot all the symmetrical gaits performed by dogs and recorded by Hildebrand [1], as well as the "C-shaped" contour that he thought captured the scope of locomotory behaviour most meaningfully. Overlaid on these data are all the feasible convergent results from every condition studied in Fig 9. Color represents relative cost: that is, the deviation of the cost for a given solution from the minimal cost at the same speed. Each data point is a complete optimization from a single random guess, resulting in a solution that satisfied all dynamics and constraints, and where successful convergence towards local optimality was indicated by the NLP solver.

The vast majority of symmetrical solutions, as well as the lowest cost, lie within a similar "C-shape" to that observed by Hildebrand. Infrequently, sub-optimal but feasible solutions are discovered outside the range observed by Hildebrand. Their presence indicates that such solutions are possible– so there seems to be no plausible physical constraint to prevent dogs from entering that region of gait space– but their relative high cost indicates that they are unlikely to be chosen from an optimization process.

In contrast, the solutions along the C contour are frequently discovered by the optimization process. All are local optima; solutions that the optimizer converged on. The remarkable similarity between the distribution of optimal solutions and Hildebrand's empirical observations suggests that dogs are also (locally) optimizing cost of transport when selecting gait.

Notably, Hildebrand's results combined observations from dog breeds of varying sizes

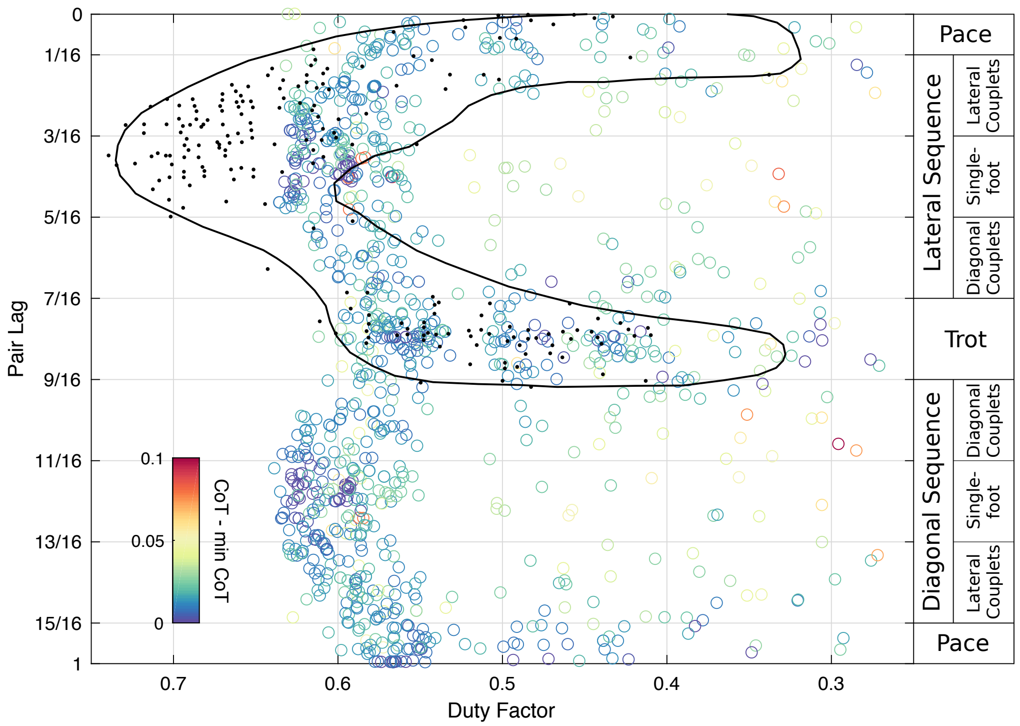

Fig 11. Symmetrical gaits discovered by the model compared to all symmetrical gaits in dogs observed by Hildebrand [1]
Data points (black dots) and contour (line) are from Figs 1 abd 2 of [1] respectively, representing mean duty factor and Pair Lag for symmetrical gaits in dogs. These are overlaid on all locally optimal symmetrical solutions (coloured circles) discovered by the model for the Belgian Malinois dataset (Fig 9). The empirical contour takes on a "C" shape in the upper (lateral sequence) region of the plot. The optimal solutions take on two "C" shapes, one in the upper half and one in the lower, as lateral and diagonal sequence gaits have equal cost in a planar model. While there is some discrepancy in the lowest duty factor, there is substantial overlap between Hildebrand's contour and the clustering of locally optimal solutions in the model. Each coloured data point represents one solution from a different random guess at any of the speeds represented in Fig 8. Colour represents cost relative to the minimal cost solution at that given speed. Gait definitions, according to Hildebrand [2], are shown on right.

and shapes (from Basset hounds to Great Danes [1]), yet the simulations used only one morphological set, based on Belgian Malinois. The consistency between simulations and empirical data, despite the differences in size, is due to dynamic similarity. While a small and large dog may move differently at the same absolute speed, they are moving at different dimensionless speeds (the square root of Froude numbers, Eq 28) and so have different dynamic constraints and opportunities. Yet when their dimensionless speed is similar, their behaviour is similar [3]. As our simulation probed a large range of Froude numbers, we explored the scope of dynamic regimes that dogs (of all *sizes*) might be expected to experience in level, steady movement.

Harder to explain is the similarity between our results and Hildebrand's despite differences in morphological *shape*. The similarity seems to suggest that general shape (at least as far as limb length to body length ratios and mass distribution) has little effect on the optimality of trotting, pacing, and single foot walking over other symmetrical gaits. The effect of morphology on the optimality of gait is something that could be explored with the present model by systematically varying morphological inputs.

One major discrepancy between the empirical and simulation results is the presence in the latter of a lower "C" that is a reflection of the first, upper "C". In this planar model there is no energetic distinction between a pair lag of p and $p \pm 0.5$, so it should come as no surprise that for every optimal gait in the upper region, there is an equally optimal gait in the lower region with the same duty factor but with a phase increase of 0.5. However, the discrepancy opens the question of why dogs (and most other quadrupedal mammals [4, 5]) use symmetrical gaits with pair lag less than 0.6 as opposed to higher pair lags. This is a question that a planar model cannot answer, but may be explained by considering full three-dimensional dynamics.

References

1. Hildebrand M. Symmetrical Gaits of Dogs in Relation to Body Build. J Morphol. 1968;124(3):353–359.

2. Hildebrand M. Analysis of Tetrapod Gaits: General Considerations and Symmetrical Gaits. In: Herman RM, Grillner S, Stein P, Stuart D, editors. Neural

Control of Locomotion. vol. 18 of Advances in Behavioral Biology. Plenum Press New York; 1976. p. 203–236.

3. Alexander RM, Jayes AS. A Dynamic Similarity Hypothesis for the Gaits of Quadrupedal Mammals. J Zool. 1983;201(1):135–152. doi:10.1111/j.1469-7998.1983.tb04266.x.

4. Lemelin P, Schmitt D, Cartmill M. Footfall Patterns and Interlimb Co-Ordination in Opossums (Family Didelphidae): Evidence for the Evolution of Diagonal-Sequence Walking Gaits in Primates. J Zool. 2003;260(4):423–429. doi:10.1017/S0952836903003856.

5. Loscher DM. Kinematische Anpassungen zur Kollisionsreduktion im Schritt vierfüßiger Lauftiere [Dr. rer. Nat.]. Freie Universität Berlin. Berlin, Germany; 2015.
