# Supplementary material for: An inelastic quadrupedal model discovers four-beat walking, two-beat running, and pseudo-elastic actuation as energetically optimal
Source: PLoS Comput Biol. 2019 Nov 21;15(11):e1007444. doi: 10.1371/journal.pcbi.1007444 (PMC6871776; doi:10.1371/journal.pcbi.1007444)
Supplement: S1 Table — (PDF) [file pcbi.1007444.s003.pdf]

Variable Bounds

	Initial		Intermediate		Final	
	**Lower**	**Upper**	**Lower**	**Upper**	**Lower**	**Upper**
t'	0	0	0	1	1	1
**STATES**						
x'	0	0	$-D'$	$2D'$	D'	D'
y'	0	$4\max(l_{i\max})$	0	$4\max(l_{i\max})$	0	$4\max(l_{i\max})$
θ	$-\pi/2$	$\pi/2$	$-\pi/2$	$\pi/2$	$-\pi/2$	$\pi/2$
$\dot{x}', \dot{y}'$	$-4D'$	$4D'$	$-4D'$	$4D'$	$-4D'$	$4D'$
$\dot{\theta}$	-4	4	-4	4	-4	4
F'_{ijT}	0	10	0	10	0	0
F'_{ijL}	0	0	0	10	0	10
$\int_0^{t'} F'_{ijL}(\tau)\,\mathrm{d}\tau$	0	0	0	10	0	10
**PARAMETERS**						
f'_{ijT}	$-\max(l_{i\max}) - D' - 1$	$\max(l_{i\max}) + D' + 1$	-	-	-	-
**CONTROLS**						
$\dot{F}'_{ijk}$	-100	100				
p'_{ijk}, q'_{ijk}	0	20	0	20	0	20
s'_{aijk}, s'_{bij}, s'_{cijk}	0	20	0	20	0	20
