# Supplementary material for: An inelastic quadrupedal model discovers four-beat walking, two-beat running, and pseudo-elastic actuation as energetically optimal
Source: PLoS Comput Biol. 2019 Nov 21;15(11):e1007444. doi: 10.1371/journal.pcbi.1007444 (PMC6871776; doi:10.1371/journal.pcbi.1007444)
Supplement: S2 Table — (PDF) [file pcbi.1007444.s004.pdf]

Main Algorithm Settings

		Major Iteration				
		1	2	3	4	
	**Input Guess**	Uniform Random Distribution	Iteration 1 output	Iteration 2 output	Interation 3 output	
	***pq* Complementarity**	Enforced by Constraint	Augmented objective (c_2=1e-3)	Augmented objective (c_2=1e-3)	Augmented objective (c_2=1e-3)	
	**Relaxation Parameter Coefficients**	[0 0 0] $\cdot$ [s_{aijk}, s_{bij}, s_{cijk}]	[100 10 10] $\cdot$ [s_{aijk}, s_{bij}, s_{cijk}]	[1000 100 100] $\cdot$ [s_{aijk}, s_{bij}, s_{cijk}]	[1000 100 100] $\cdot$ [s_{aijk}, s_{bij}, s_{cijk}]	
**SNOPT**	**Max Iter**	500	2000	2000	2000	
	**Tol**	1e-06	1e-08	1e-08	1e-08	
**MESH**	**Max Iter**	1	2	3	8	
	**Other settings (all major iterations)**	AutoScaling: Automatic-hybrid update	Method: RPM-integration	Derivatives: Sparse CD	Mesh Tol: 1e-4	Initial mesh: 4 mesh intervals with 4 collocation points each
