# Supplementary figures and images for: An inelastic quadrupedal model discovers four-beat walking, two-beat running, and pseudo-elastic actuation as energetically optimal
Source: PLoS Comput Biol. 2019 Nov 21;15(11):e1007444. doi: 10.1371/journal.pcbi.1007444 (PMC6871776; doi:10.1371/journal.pcbi.1007444)

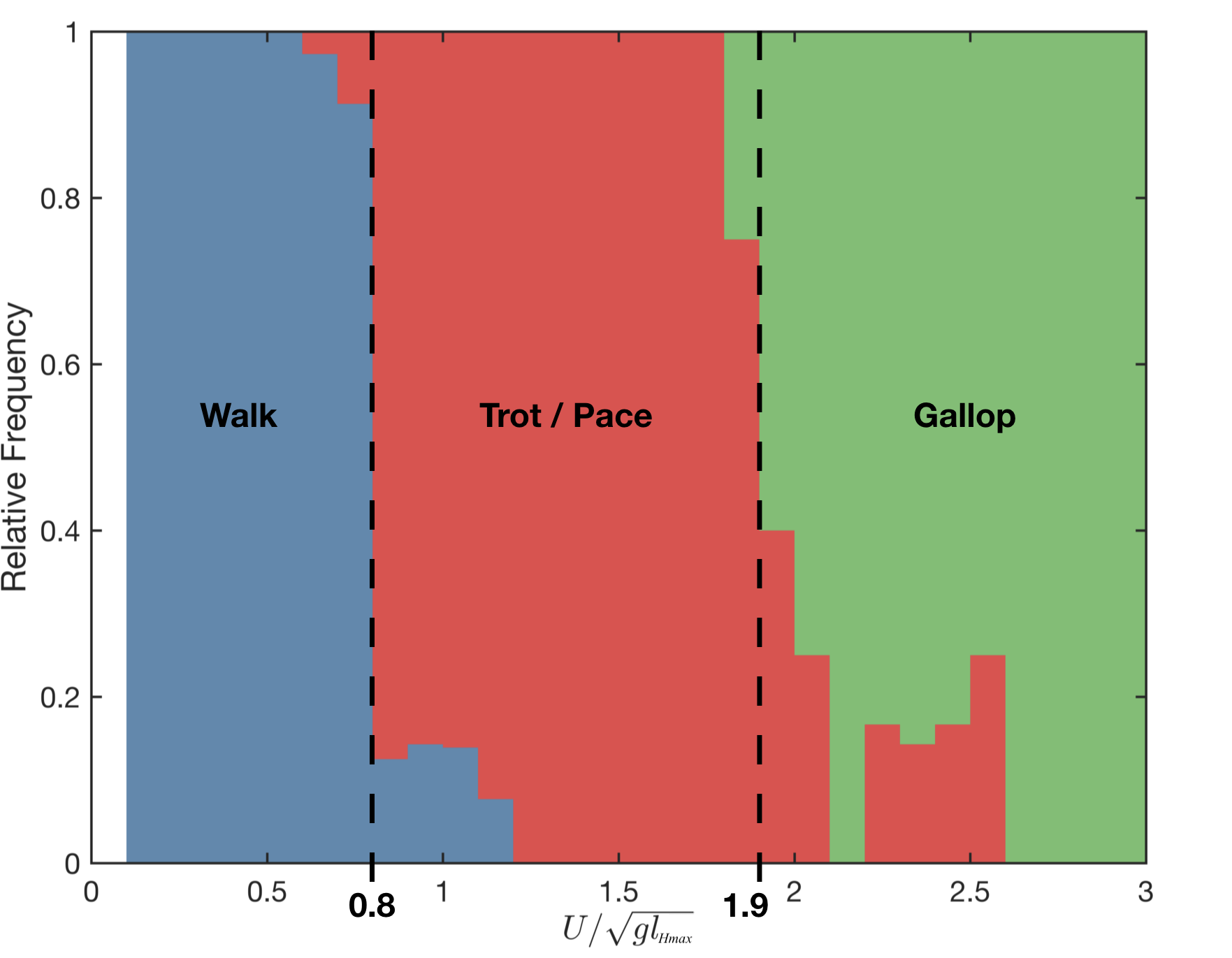

Supplement: S1 Fig — Walking shown in blue, trotting/pacing in red and galloping in green. Observations normalized to total count in each bin. Data from Fig 4 in [40]. (TIF) [file pcbi.1007444.s005.tif]
